# Impact of multifactorial interventions with medication and lifestyle optimization on patients with type 2 diabetes: A randomised controlled trial

**DOI:** 10.1371/journal.pone.0327211

**Published:** 2025-07-09

**Authors:** Marwan El-Deyarbi, Luai Ahmed, Jeffrey King, Zelal S. Adi, Ahmed Al Juboori, Nirmin A. Mansour, Huda Al Nuaimi, Rami Beiram, Salahdein Aburuz

**Affiliations:** 1 Department of Pharmacology, College of Medicine and Health Sciences, United Arab Emirates University, Al Ain, UAE; 2 Department of Pharmacy, Oud Al-Touba Diagnostic and Screening Clinic, Ambulatory Health Services, Abu Dhabi Health Services Co. (SEHA), Al Ain, UAE; 3 Institute of Public Health, College of Medicine and Health Sciences, United Arab Emirates University, Al Ain, UAE; 4 David Geffen School of Medicine at the University of California, Los Angeles, California, United States of America; 5 Department of Geriatrics and Extended Care, VA Greater Los Angeles Healthcare System, U.S. Department of Veterans Affairs, Los Angeles, California, United States of America; 6 Division of Endocrinology, Oud Al-Touba Diagnostic and Screening Clinic, Ambulatory Health Services, Abu Dhabi Health Services Co. (SEHA), Al Ain, UAE; 7 Clinical Nutrition and Dietary Department, Oud Al-Touba Diagnostic and Screening Clinic, Ambulatory Health Services, Abu Dhabi Health Services Co. (SEHA), Al Ain, UAE; Tehran University of Medical Sciences, IRAN, ISLAMIC REPUBLIC OF

## Abstract

**Background:**

Clinical evidence on the protective effects of a balanced diet, exercise, and medication adherence along with intensive glucose-lowering therapies on diabetes progression is lacking, and interventions that are most effective in slowing cardiorenal metabolic complications in patients with diabetes remain unelucidated.

**Objective:**

To determine the effects of long-term multifactorial interventions on clinical outcomes in Emirati patients with diabetes attending ambulatory healthcare clinics.

**Methods:**

We conducted a randomised controlled clinical trial at the Oud Al-Touba Clinic involving 192 participants with diabetes, who were blinded to the intervention and control groups, and followed up for 1 year. At the 3-, 6-, and 9-month visits, the intervention and control groups received multifactorial interventions and standard routine care, respectively. Glycated haemoglobin A1c (HbA1c) levels, estimated glomerular filtration rate (eGFR), blood pressure, electrolyte levels, and cardiovascular events were assessed at study completion.

**Results:**

During a mean follow-up of 11.9 months, 40.4% of the participants in the intervention group (31.6% in the control group) achieved diabetes control (HbA1c < 7%), with a significant mean difference of −0.36% in HbA1c levels between the groups (95% CI: −0.54 – −0.19, P < 0.01). Participants in the multifactorial group achieved a significant mean difference in low-density lipoprotein cholesterol levels (mean difference = −0.14, 95% CI: −0.27–0.001, P < 0.03), and significant adjusted mean difference of eGFR levels difference (3.93 mL/min/1.73 m^2^, 95% CI: 1.27–6.58, P < 0.01) at study completion compared to those in the control group. Moreover, the percentage of participants in the intervention group who met the blood pressure target increased from 38.3% to 51.1%, accompanied with a decrease in serum electrolyte levels, compared to 34.7% to 36.7% in the control group at the end of the follow-up.

**Conclusions:**

Implementing multifactorial interventions by a multidisciplinary team improved several clinical manifestations, including HbA1c, SBP, and eGFR, and decreased cardiovascular risk factors despite the decreased diabetes medication use.

**Trial registration:**

ClinicalTrials.gov NCT04942119

## Introduction

The prevalence of type 2 diabetes mellitus (T2DM) is increasing worldwide, with the greatest surge in low- and middle-income countries [1]. The Middle East and North Africa have the highest prevalence of type 2 diabetes, with approximately 12.2% of the adults diagnosed with the disease [2]. It is associated with both macrovascular and microvascular complications, as well as higher diabetes treatment costs due to the increased prevalence of risk factors and complications-related mortality [3–4]. Diabetic kidney disease (DKD) is the leading cause of end-stage kidney disease and substantially contributes to cardiovascular events [3].

Long-term uncontrolled hyperglycaemia in patients with diabetes is a significant risk factor for both microvascular and macrovascular complications [5]. Moreover, the prevalence of DKD has increased to 33.1% among adults with diabetes [6], with increased risk of mortality and greater healthcare costs [7]. Over the last few years, major advances in surrogate markers of diabetic and renal complications have been made to reduce the disease burden on patients and healthcare systems. However, evidence for improvement in diabetes-related illnesses and patients’ health-related quality of life is scarce [8–10].

Although evidence demonstrates the benefits of individual interventions in reducing the burden of chronic T2DM and DKD, the most effective multifactorial intervention remains unclear. Such interventions are likely to involve intensive serum electrolyte panel monitoring [11], low-glycaemic index diets with high fiber content to improve glycated haemoglobin A1c (HbA1c) levels in patients with T2DM [12–13], pharmacist-led medication therapy management (MTM) [14], and the use of telemedicine technology through mobile applications [15]. These factors have long-term effects on diabetes, DKD progression, and hard endpoints such as cardiovascular events and all-cause mortality [16]. However, the effect size of the combination of these interventions has not yet been clearly elucidated.

Moreover, dietary sodium (Na), potassium (K), and magnesium (Mg) consumption in the management of patients with diabetes has been assessed in limited studies [17–20]. Electrolyte disorders such as Na, K, and Mg deficiencies are shared features of T2DM, suggesting that serum electrolytes such as K and Mg can be considered indicators of DKD progression and play an important role in its treatment [21–22]. In addition, higher rates of cardiovascular events including hypertension, congestive heart failure, and all-cause mortality, have been associated with lower Na intake in patients with diabetes [23–25],

Consequently, guidelines from the Institute of Medicine and the American Diabetes Association recommend an upper limit of daily Na, K, and Mg intake of 2300 mg [26–27], 4680 mg [26], and 320–400 mg [28], respectively.

Although dietary Na does not affect blood glucose levels, a meta-analysis revealed that participants with low Na intake had significantly higher blood glucose levels than those with normal or high Na intake [29]. Furthermore, dietary Na is a modifiable risk factor for cardiovascular diseases such as hypertension, a leading cause of morbidity and mortality in patients with diabetes [30].

In contrast, inappropriate dietary K intake is a cause of hypokalaemia in these patients [31], and it is reportedly associated with the development of hyperglycaemia by disrupting the function of ATP-sensitive K channels in pancreatic islets, leading to impairment of K-dependent insulin release in response to glucose overload [32]. Not surprisingly, hypokalaemia is the most common electrolyte disturbance associated with poor glycaemic control in clinical practice.

Numerous studies have highlighted that chronic hypokalaemia can lead to several renal abnormalities and dysfunctions, which are reversible with K repletion. Chronic K depletion may contribute to hypokalaemic nephropathy, which produces nonspecific vascular lesions in the epithelial cells of the proximal tubule and occasionally in the distal tubule [33]. This abnormality generally develops in one month and is readily reversible with K repletion.

The effects of low serum Mg levels on diabetes are explained by its role in mediating insulin secretion and impacting insulin signalling and resistance, with similar effects on prediabetes progression [34]. An increasing number of studies have found that hypomagnesaemia is reportedly uncommon in patients with uncontrolled diabetes mellitus and is involved in the progression of diabetes [35]. It is also associated with increased urinary Mg excretion which is reversed by correction of hyperglycaemia [36–37], and dietary Mg intake plays a role in modifying the process [38]. These findings suggest that hypomagnesaemia may impair glucose excretion, increasing the risk of T2DM, which may play a role in the pathogenesis of some diabetes complications [39]. However, further studies are required to assess this association between hypomagnesaemia and the development of diabetes.

Additionally, the relationship between dietary Na and K intake and chronic kidney disease in the general US population has been examined in some studies, indicating that a higher intake of Na and K is associated with lower odds of developing chronic kidney disease among US adults [40]; however, this hypothesis remains debatable. Moreover, Shahid and Mahboob previously observed an electrolyte imbalance during DKD progression in patients with diabetes compared with those without diabetes or hypertension [41]. Another study showed significantly reduced Na, and Mg levels compared to those in patients with euglycemia [42].

In this study, a nutritional intervention with a healthy diet plan directed at optimising electrolyte levels was included, which has been proven to be a cornerstone in preventing and treating diabetic complications, including progression to DKD [43]. Being overweight or obese affects disease management, hinders blood glucose control in patients with diabetes [44], and significantly impacts various health outcomes and health-related quality of life compared with patients with normal weight [45].

Other studies have investigated the correlation between pharmacist-led interventions through medication counselling and the improvement of clinical outcomes and medication adherence in patients with T2DM [46–47]. Moreover, the use of technology and telemedicine in disease management has proven beneficial for patients with diabetes and other chronic diseases, demonstrating a significant reduction in mortality rates [48]. The use of mobile applications can enhance efficiency, enable patients to manage their health more effectively [49], and serve as a useful tool in the treatment of patients with complex diseases who require more frequent visits to achieve improved glycaemic control or face difficulties in accessing the healthcare system [50].

The effect of a multifactorial intervention approach involving nutritional and exercise counselling, pharmacist-led MTM, and optimisation of serum electrolyte levels on diabetic clinical outcomes, including glycaemic control, kidney function, and serum electrolyte levels, was investigated in this study in Emirati patients diagnosed with T2DM in an outpatient setting.

The primary objectives were as follows: (1) to measure the mean change from baseline in HbA1c levels, blood pressure (BP), and lipid profile in the multifactorial intervention group compared to the control group. The secondary objectives were as follows: (1) to evaluate changes in serum electrolyte levels, including serum Na, K, and Mg levels, from baseline in both groups; (2) to assess changes in estimated glomerular filtration rate (eGFR), creatinine, and serum albumin in both groups; and (3) to identify cardiovascular and drug adverse events during the follow-up period.

## Materials and methods

### Study design

This single-center, randomized, controlled, multidisciplinary trial was conducted at the Oud Al-Touba Diagnostic and Screening Clinic, an ambulatory healthcare center in Abu Dhabi, United Arab Emirates, and involved a 12-month follow-up. The trial protocol was registered with the US National Institutes of Health (ClinicalTrials.gov protocol registration: NCT04942119).

### Ethics and dissemination

This study was approved by the SEHA Research Oversight and Ethics Committee of the United Arab Emirates (approval number: SEHA-IRB-021). At their office, the physician discussed the possible participation of eligible subjects in the study, and if satisfied, the participant signed the written consent form and received a copy along with the study information sheet. Patient confidentiality was maintained during the study, and all de-identified protected health information was recorded electronically and saved securely using password protection, limiting access to authorised research personnel and the SEHA Research Oversight and Ethics Committee for auditing and monitoring purposes.

The trial results were communicated to the ethics committee in the final report, and physicians from the Oud Al-Touba clinic had access to the clinical trial protocol and research results while maintaining patient confidentiality and advancing medical research and understanding. The authorship agreement declaring the intellectual contributions of the research team, the origin of the research, and the accountability of any published information was written and reviewed by the research team before publication.

### Study population

**Fig 1:**
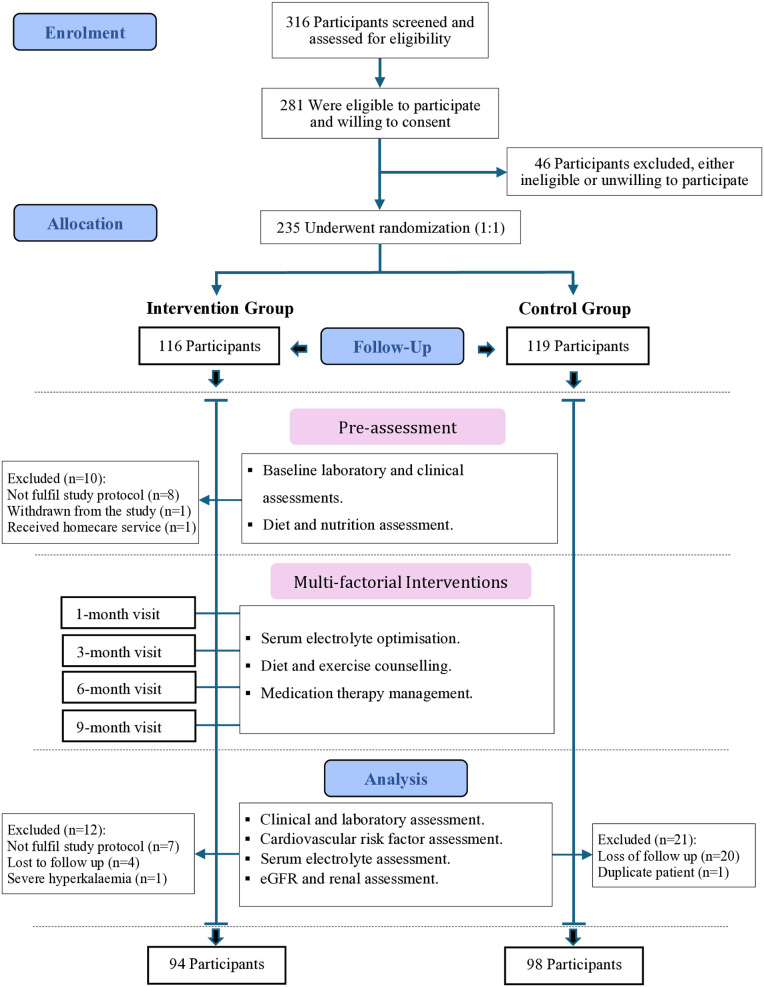
Study participants flow diagram. Na, Sodium; K, Potassium; Mg, Magnesium; MTM, Medication therapy management; eGFR, estimated glomerular filtration rate.

Between the 9th of July and the 28th of September 2021, 316 patients were screened for eligibility from endocrinology or chronic disease clinics, and 281 eligible patients were invited to participate. Only 235 patients consented to participate in the study and were randomised into either the intervention (116 participants) or the control group (119 participants) (Fig 1).

A total of 192 Emirati patients previously diagnosed with T2DM [51] who had been taking the same diabetes medications for 12 weeks prior to the study and whose electronic health records were available on Salamtak (Oracle Health, Oracle Corporation) were included in the analysis. Those with diabetic macrovascular complications, a medical history of cancer, chronic liver disease, or high cardiovascular risk [defined as a 10-year predicted atherosclerotic cardiovascular disease (ASCVD) risk of ≥ 20%, calculated either using Framingham Risk Score or ASCVD Risk Estimator Plus] within the last 12 months were excluded, as they require individualised risk assessment and therapy management [52–53].

*Inclusion criteria:*

- Male or female Emirati patients aged 30–65 years.
- Patients with an HbA1c level of at least 7.0% with stable glucose-lowering medication recorded in Cerner for at least 12 weeks before recruitment.
- Patients with normal to moderately impaired renal function, defined as an eGFR of >30 mL/min/1.73 m^2^, for >3 months at baseline (stages G1, G2, G3a, and G3b of chronic kidney disease) [54].

*Exclusion criteria:*

- Severe or symptomatic hypo- or hypernatremia, hypo- or hyperkalaemia and/or hypo- or hypermagnesemia, metabolic acidosis, or hypophosphataemia, with or without proximal renal tubular acidosis and Fanconi syndrome.
- Prolonged hypokalaemia with surreptitious diuretic use, laxative abuse, eating disorders, or primary aldosteronism.
- Receiving medications that may cause drug-induced acute renal failure during the observation period and may be implicated in hypomagnesaemia (e.g., aminoglycoside antibiotics, cyclosporine, amphotericin B, cisplatin, pentamidine, and foscarnet).
- Undergone bariatric surgery within the past two years or other gastrointestinal surgeries that induce chronic malabsorption.
- Premenopausal women who are nursing or were pregnant within the last 12 months.
- Presence of chronic illnesses such as liver disease, cancer, and other uncontrolled endocrine disorders or diabetic macrovascular complications.

A computer-generated random number was used for randomising participants using Microsoft Excel to generate allocation sequences by the principal investigator for each participant in both groups, and allocation concealment was executed by sequential numbering, signifying their enrolment in the study. Refusal to participate in the study for any reason, such as absence of desire or motivation, cultural barriers, or privacy concerns was recorded.

After enrolment, 22 participants were excluded from the intervention group because they either withdrew from the study or had missing follow-up data, and 21 participants were excluded from the control group because of missing laboratory tests by study investigators at the end of the study period. Consequently, 192 participants (94 and 98 in the intervention and control groups, respectively) were included in the analysis at the conclusion of the follow-up period after 12 months. The study ended with the last patient follow visit in the intervention group on 23rd of September 2022.

### Study procedures

#### Baseline laboratory and clinical assessments.

Each participant underwent individual clinical and laboratory assessments at the initial visit and one year after randomisation or at study exit, in addition to medication adherence assessment using a validated questionnaire [55] and diet and exercise assessment by a specialised dietitian using validated tools.

Laboratory and clinical assessments included BP measurement and documentation of laboratory data for each eligible participant at the initial visit; if no updated laboratory results from the preceding three months were included in the medical record, participants were scheduled for blood sample collection in the clinic, and the same procedure was carried out once the 12-month follow-up period was concluded. Blood samples were tested at the Abu Dhabi Health Services Company (SEHA) Central Laboratory. Laboratory tests included HbA1c level, low-density lipoprotein cholesterol (LDL-C) level, complete blood count, electrolyte panel including K and Mg levels, eGFR, serum creatinine level, and albumin/creatinine ratio. BP measurements and laboratory tests were performed according to the standard procedures for BP measurement and blood sample withdrawal at the clinic.

According to American Diabetes Association guidelines [56], participants were considered on target if they achieved HbA1c and BP goals of <7% (on treatment with antihyperglycemic medication) and <130/80 mmHg, respectively.

Moreover, according to the 2018 cholesterol guideline and American College of Cardiology recommendations [57], moderate-intensity statin therapy is recommended (without calculating 10-year ASCVD risk) for patients with diabetes mellitus aged 40–75 years with an LDL-C of ≥1.8 mmol/L. Considering that the mean age and mean LDL-C of the study population were 60 years and ≥3 mmol/L, respectively, and 65% of the participants were on statin therapy (with or without ezetimibe or a PCSK9 inhibitor) at study enrolment, a cutoff point of LDL-C < 1.8 mmol/L was established as the target for this study.

At the initial visit, both groups completed a pre-assessment nutrition knowledge questionnaire with eight questions evaluating knowledge of the importance of frequent laboratory blood tests; regular monitoring of kidney function; recommended dietary allowances of Na, K, and Mg; food sources of these elements; and symptoms of deficiency S4 and S5 Tables. Moreover, they received educational information based on dietitian evaluation as part of routine care in the clinic.

### Standard routine care for the control group

During the study period, the control group received the standard routine care provided at the clinic, including routine follow-ups and clinical assessments (as needed). It also included a follow-up schedule every 3 months with the treating physician, vital signs monitoring at the visited clinic, physician consultation, investigational or laboratory tests requested by the treating physician depending on the condition of the patient, patient counselling at the pharmacy counter, dietitian counselling service if requested by the patient or referred by the treating physician, and a call reminder from the clinic call centre three days before the appointment.

Moreover, HbA1c levels of the control group were recorded at least twice yearly (at initial and exit visits) in participants with stable glycaemic control who met the treatment goals and quarterly in those not meeting the goals, at the discretion of the attending physician [58]. Other laboratory tests were also performed biannually (or more frequently at the request of the physician), and any major adverse cardiac events, emergency room visits, or hospitalisations were recorded and compared with the baseline data.

### Multifactorial interventions for the intervention group

The intervention group received the following multifactorial interventions with assessments at the initial visit and at 3-, 6-, and 9-month visits after study enrolment. They were instructed to report any possible side effects or factors that may affect dietary and medication adherence, as well as any lifestyle or dietary modifications during the study.

#### Serum sodium, potassium and magnesium optimization.

During the follow-up, the intervention group was evaluated for electrolyte imbalance, particularly Na, K, and Mg levels. The metabolic panel (including serum Na, K, and Mg levels) and eGFR were measured at baseline and at 3, 6, 9, and 12 months after recruitment.

Participants were evaluated for symptoms of electrolyte imbalance. In patients with no or minimal symptoms of mild to moderate hyper-, or hyponatremia [59–60], hypomagnesaemia [61] and/or hyper- or hypokalaemia [31,62], treatment is initiated based on the recommended clinical pathway with the primary goal of preventing or treating life-threatening complications while diagnosing and treating the underlying disease. Patients with abnormal levels of Na, K, or Mg resulting in severe symptoms were withdrawn from the study [63].

#### Diet and exercise counselling.

Participants in the intervention group received structured diet and exercise counselling by a registered dietitian during the follow-up visit to the clinic and every three months, as described in detail elsewhere [64]. Participants also received monthly follow-up phone calls after each follow-up visit by the same dietitian to reinforce optimal diet adherence, balance caloric and mineral intake, and ensure effective lifestyle modifications through exercise.

The revised Summary of Diabetes Self-Care Activities scale [65] was used for diet and exercise assessment in the intervention group during each follow-up visit. The original Summary of Diabetes Self-Care Activities post-assessment questionnaire was expanded to include two additional questions measuring adherence to prescribed nutritional supplements, including prescribed Mg and K supplements by the physician, and adherence to periodic scheduled laboratory tests. Participants completed quarterly questionnaires to assess their adherence to a structured diet and ascertain their continuation of usual physical activity at each follow-up visit [64].

#### Medication therapy management (MTM).

Each participant in the intervention group was enrolled in the MTM program with private counselling and medication adherence assessment by a clinical pharmacist, as described in in detail elsewhere [66]. MTM encompasses several activities, including comprehensive medication reconciliation and reviews to identify and resolve medication-related issues or adverse events, modifying or optimising medication therapy in coordination with healthcare providers, evaluating or recommending necessary laboratory tests, and formulating a pharmaceutical care plan to evaluate and monitor patient adherence and response to therapy. Moreover, other services, such as patient medication booklets and SEHA mobile application education, were provided during the MTM program to improve adherence to therapeutic regimens and optimise clinical outcomes.

Medication adherence was measured at baseline and each follow-up visit using a validated questionnaire and by calculating the fixed medication possession ratio, adjusted for medication change or hospital stay during the study, in reference to the medication refill records of the patients from both private and governmental pharmacies recorded on the Salamtak EHR platform [66].

### Study outcomes

The primary outcomes assessed in the study were (1) changes in HbA1c levels, BP, and lipid profile in the intervention group compared to the control group. In addition, secondary outcomes included (1) the difference in serum electrolyte levels, including Na, K, and Mg serum levels from baseline; (2) the change in eGFR, urine albumin-creatinine ratio, and the incidence or worsening of nephropathy (increase in serum creatinine level) from baseline; and (3) the incidence of drug adverse events and cardiovascular events during the follow-up period.

### Statistical analysis

The sample size was calculated using the Giga sample size online calculator [67], with a power of 80% and an alpha level of 5% to detect a minimal difference of 0.5% in HbA1c levels between groups, with a mean HbA1c of 7.7 ± 1.3 (mean ± SD) at baseline (for 50 participants). A total of 84 participants per group were required for the primary outcome, and a sample size of 184 participants was calculated considering a 10% expected dropout rate during the follow-up period.

Both groups were tested for normality using the Shapiro–Wilk test. Demographic data and clinical factors of the participants are presented as percentages for categorical variables, using chi-squared tests, and as means (± SD) for continuous variables (e.g., age, HbA1c, eGFR), analysed using Student’s t-test and analysis of covariance (ANCOVA) with Bonferroni adjustment for multiple comparisons. Analysis of covariance of the difference in change in eGFR at study exit was conducted taking into consideration age, sex, BMI, and mean HbA1c, SBP, DBP, Na, K, and Mg levels as covariates, using regression-based covariates scores (3 scores used, one score for age, sex and BMI, one score for mean HbA1c, SBP, and DBP, and one score for mean Na, K, and Mg). Analyses were performed using IBM SPSS Statistics (version 26), and differences were considered significant at p < 0.05.

### Data management

Each patient was assigned a code documented in the consent form by the principal investigator and another assigned researcher, and this information was saved in a password-protected file. All patient identification data were removed to conceal randomisation and minimise the predictability of the generated random sequence.

Quality control plans for data management were also implemented. Clearly formatted data-collection forms were designed to encourage the collection of high-quality data. Laboratory reference ranges were integrated into the Salamtak EHR platform with predefined upper and lower normal levels and integrated alerts for any abnormal test results, thereby enabling more accurate data collection. For any missing laboratory or dietitian appointments, a new appointment was scheduled after calling the patient to confirm the new date and documenting the reasons for noncompliance in the progress note.

### Rules for early stopping

Participants had the right to withdraw consent, stop or postpone the assessment interviews, or fill out questionnaires if they became distressed at any time during the study period. Furthermore, the participants exited the study if they visited the emergency department, were hospitalised because of renal or cardiac problems, or developed severe or symptomatic electrolyte disorder or metabolic acidosis.

## Results

### Demographic data

The patient demographics and baseline clinical and laboratory data are presented in Table 1. In both groups, demographic data and baseline clinical characteristics were comparable, with no significant differences observed. The mean age of the participants was approximately 60 years, with a mean body mass index of 31, and 60% of them were women (Table 1).

**Table 1:** Characteristics of study participants at baseline.

Patients’ Characteristics	Control	Intervention	P Value
	n = 98	n = 94	
**Demographic**			
Age – year	61.8 ± 13.9	59.6 ± 11.7	0.89
BMI	31.3 ± 7.2	30.5 ± 8.6	0.66
Sex – female	58 (69.2)	67 (72.8)	0.40
Marital status – married	76 (77.6)	75 (79.8)	0.72
**Baseline clinical data**			
Duration of diabetes – yr, median (IQR)	24.5 (16-30)	25.5 (17-34)	0.15
HbA1c before intervention	7.7 ± 1.3	7.4 ± 1.4	0.54
Insulin therapy	11 (3.9)	16 (5.7)	0.64
Non-insulin therapy	87 (88.7)	78 (82.9)	0.72
SBP	140.3 ± 10.5	136.2 ± 13.9	0.77
DPB	87.6 ± 10.0	85.6 ± 9.3	0.85
LDL cholesterol	3.2 ± 1.1	3 ± 1.3	0.53
eGFR	88.5 ± 26.1	90.4 ± 21.4	0.20
Sodium	141.5 ± 4.1	139.3 ± 2.5	0.49
Potassium	4.3 ± 0.4	4.28 ± 0.6	0.07
Magnesium	0.79 ± 0.1	0.8 ± 0.1	0.11
**Initial assessment data**			
Patients on glycaemic target	35 (35.7)	38 (40.4)	0.50
Patients on BP target	34 (34.7)	36 (38.3)	0.61
Patients on intensive LDL-C target	18 (18.4)	21 (22.3)	0.49
Cardiovascular disease	2 (2)	4 (4.1)	0.36
eGFR below 60	13 (13.3)	12 (12.8)	0.22

Data are M ± SD or n (%); BMI, Body mass index (kg/m^2^); eGFR, Estimated glomerular filtration rate (mL/min/1.73 m^2^); HbA1c, Glycated haemoglobin A1c (%, NGSP); LDL, Low-density lipoprotein (mmol/L); SBP, Systolic blood pressure (mmHg); DPB, Diastolic blood pressure (mmHg).

As shown in Table 1, 40.4% of the participants in the intervention group achieved target HbA1c with a mean baseline of 7.4%, 37% achieved their BP goal with mean systolic BP (SBP) and diastolic BP (DBP) of 136.2 mmHg and 85.6 mmHg, respectively, and only 22.3% of the participants achieved LDL-C target with a mean LDL-C level of 3 mg/dl. In the control group, the mean HbA1c was 7.7% at baseline, with fewer participants achieving the HbA1c target (35.7%), higher SBP and DBP means (140.3 mm Hg and 87.6 mm Hg respectively), and a mean LDL-C level of 3.2 mg/dl. The detailed characteristics of the medications used in each treatment regimen for both treatment groups are presented in Table 2.

**Table 2:** Characteristics of the medications in each regimen at baseline.

Medications in each regimen	Control		Intervention	
	Baseline	Study exit	Baseline	Study exit
	n = 541	n = 556	n = 516	n = 506
**Antihyperglycaemic medication**	274 (50.6)	280 (50.4)	253 (49)	249 (49.2)
Insulin	11 (4)	13 (4.6)	16 (6.3)	17 (6.8)
GLP	42 (15.3)	44 (15.7)	34 (13.4)	39 (15.7)
Metformin	86 (31.4)	83 (29.6)	84 (33.2)	84 (33.7)
Sulfonylurea	36 (13.1)	34 (12.1)	32 (12.6)	28 (11.2)
TZD	6 (2.2)	5 (1.9)	11 (4.3)	10 (4)
SGLT2	60 (21.9)	64 (22.9)	49 (19.4)	52 (20.9)
DPP4 inhibitor	33 (12)	37 (13.2)	27 (10.7)	19 (7.6)
**Antihypertensive drugs** ^¥^	152 (28.1)	163 (29.3)	142 (27.5)	138 (27.3)
Renin-angiotensin	65 (42.8)	69 (42.3)	60 (42.2)	63 (45.9)
Beta-blockers	18 (11.8)	19 (11.7)	26 (18.3)	24 (17.5)
Ca-channel blockers	42 (27.6)	47 (28.8)	36 (25.3)	35 (25.5)
Diuretics	22 (14.5)	21 (12.9)	17 (11.9)	15 (10.9)
Other	5 (3.3)	7 (11.1)	3 (2.1)	1 (0.7)
**Antihyperlipidaemic drugs** ^₳^	115 (21.3)	113 (20.3)	121 (23.4)	119 (23.5)
Statins	76 (66.1)	74 (65.5)	82 (67.8)	81 (68.1)
Ezetimibe	28 (24.3)	26 (23)	16 (13.2)	15 (12.6)
Omega-3	4 (3.5)	4 (3.5)	12 (9.9)	10 (8.4)
Pcsk9 inhibitors	7 (6.1)	9 (7.9)	11 (9.1)	13 (10.9)

Data are n (%); GLP-1, Glucagon-like peptide 1agonists; DPP-4, Dipeptidyl peptidase 4; Pcsk9, Proprotein convertase subtilisin/kexin type 9; SGLT2, Sodium-glucose co-transporter-2 inhibitor; TZD: Thiazolidinedione.

^¥^Intervention (n=86), Control (n=94).

^₳^Intervention (n=91); Control (n=95).

### Multifactorial intervention outcomes

#### Clinical and laboratory outcomes.

At the end of the follow-up period, the number of participants that achieved the HbA1c target of <7% was significantly higher in the intervention group as compared to those in the control group (52.1% and 34.7%, P < 0.01, respectively), with a significant mean difference in the change in HbA1C between the groups at study exit compared to baseline (−0.36, 95% CI: −0.54 – −0.19, P < 0.01) (Table 3).

**Table 3:** Clinical and laboratory measurements at study exit.

Cardiovascular risk factors	Baseline		Study exit		Mean difference^¥^ (95% CI)	P Value
	Control	Intervention	Control	Intervention		
	(n = 98)	(n = 94)	(n = 98)	(n = 94)		
**Patients on glycaemic target**	35 (35.7)	38 (40.4)	34 (34.7)	49 (52.1)	–	0.01^£^
**HbA1c**	7.7 ± 1.3	7.4 ± 1.4	7.8 ± 1.2	7.2 ± 1.2	−0.36 (−0.54 – −0.19)	<0.01^*^
**Patients on BP target**	34 (34.7)	36 (38.3)	36 (36.7)	48 (51.1)	–	0.04^£^
**Systolic blood pressure**	140.3 ± 10.5	136.2 ± 13.9	138 ± 11.6	133.8 ± 16.5	−0.003 (−1.48–1.47)	0.49
**Diastolic blood pressure**	87.6 ± 10.0	85.6 ± 9.3	86.5 ± 11.7	84.7 ± 9.7	0.17 (−0.92–1.26)	0.38
**Patients on intensive LDL-C target**	18 (18.4)	21 (22.3)	23 (23.5)	33 (31.1)	–	0.08^£^
**LDL cholesterol**	3.2 ± 1.1	3 ± 1.3	3.1 ± 1.1	2.7 ± 1.2	−0.14 (−0.27–0.001)	0.03^*^

Data are presented as mean ± SD or numbers (%); HbA1c, Glycated haemoglobin A1c (%, NGSP); LDL-c, Low-density lipoprotein cholesterol (mmol/L).

^¥^Mean difference in the change between the studied groups at study exit compared to baseline.

^£^Chi-square statistic between the study groups at study exit.

In addition, improvements in other clinical outcomes, specifically BP and LDL-C levels, were observed at the end of the study. The number of participants who had achieved the BP target at the end of the follow-up period increased in the intervention group from 38.3% to 51.1%, compared to 34.7% to 36.7% in the control group; however, the differences in mean group differences for systolic and diastolic BP readings at study exit and baseline were not significant.

Comparable results were observed in LDL-C levels, with a significant difference in mean LDL-C level between baseline and study exit across both groups (−0.14, 95% CI: −0.27–0.001, P < 0.03) (Table 3).

#### Serum electrolyte analysis (serum Na, K, and Mg levels).

The outcome analysis compared the end-of-follow-up and baseline laboratory tests of all participants. Those with the following classes of medications in their medication regimen, known to affect serum electrolyte levels (particularly Na and K serum levels), were instructed to take their medication at the time of the blood test (Table 4).

**Table 4:** Medications potentially affecting serum electrolyte levels (serum Na and K levels).

Medications in each regimen	Intervention		Control	
	Baseline	Study exit	Baseline	Study exit
**Total number of medication (n)**	**n = 516**	**n = 505**	**n = 541**	**n = 556**
**SGLT2**	49 (9.5)	52 (10.3)	60 (11.1)	64 (11.5)
**Renin-angiotensin**	60 (11.6)	63 (12.5)	65 (12)	69 (12.4)
**Diuretics** ^**¥**^	17 (3.3)	15 (2.9)	22 (4.1)	21 (3.8)

Data are presented as numbers (%); SGLT2, Sodium-glucose co-transporter-2 inhibitors.

^¥^Potassium-sparing, loop, and thiazide diuretics.

In the intervention group, serum Na levels were decreased compared to the standard of care group, and the mean concentrations (± SD) of serum Na at study exit and baseline were 138.9 ± 2.4 and 139.3 ± 2.5 mmol/L, respectively, while in the control group, they were 142.4 ± 3.2 and 141.5 ± 4.1, respectively, resulting in a significant mean difference between the groups (−1.28, 95% CI: −2.04 – −0.52, P < 0.01) (Table 5).

**Table 5:** Serum electrolyte levels between the intervention and control groups.

Electrolyte serum levels	Baseline		Study exit		Mean difference (95% CI)	P Value
	Control	Intervention	Control	Intervention		
	(n = 98)	(n = 94)	(n = 98)	(n = 94)		
**Sodium** ^¥^	141.5 ± 4.1	139.3 ± 2.5	142.4 ± 3.2	138.9 ± 2.4	−1.28 (−2.04 – −0.52)	<0.001^*^
**Potassium** ^¥^	4.33 ± 0.4	4.28 ± 0.6	4.29 ± 0.4	4.45 ± 0.5	0.22 (0.07–0.36)	0.003^*^
**Magnesium** ^¥^	0.79 ± 0.1	0.8 ± 0.1	0.78 ± 0.1	0.84 ± 0.1	0.04 (0.02–0.06)	<0.001^*^

Data are presented as Mean ± SD.

^¥^mmol/L.

*Significant p-value <0.05.

Serum K levels increased in the intervention group at study exit compared to baseline (4.45 ± 0.5 vs 4.28 ± 0.6, respectively), while the control group marginally decreased (4.29 ± 0.4 vs 4.3 ± 0.4). The mean difference in the change between the groups at the study exit compared to baseline was significant (0.22, 95% CI: 0.07–0.36, P < 0.01). Similarly, Mg levels increased in the intervention group from 0.8 ± 0.1 at baseline to 0.84 ± 0.1 at the end of the follow-up period, whereas they decreased in the control group (0.79 ± 0.1 and 0.78 ± 0.1, respectively), with a significant mean difference between the groups (0.04, 95% CI: 0.02–0.06, P < 0.01) (Table 5).

#### Renal outcomes and eGFR progression.

Using factor analysis for the possible covariates (such as age, sex, BMI, HbA1c, SBP, DBP, Na, K, and Mg levels), we conducted regression-based component score analysis in SPSS, reducing the covariates into three component scores for ANCOVA analysis which shows homogeneity of regression with no statistically significant effect between regression coefficients. In the intervention group, the kidney function, as measured by eGFR, slightly increased with a mean (± SD) value of 90.8 ± 19.8 mL/min/1.73 m^2^ compared to 90.4 ± 21.4 mL/min/1.73 m^2^ at baseline, while it decreased in the control group at study exit compared to baseline (85.5 ± 23.2 mL/min/1.73 m^2^ and 88.5 ± 26.1 mL/min/1.73 m^2^, respectively), with a significant difference in the adjusted means between the two groups for the eGFR difference at study exit compared to baseline when we control for the above covariates (3.93, 95% CI: 1.27–6.58, P < 0.01) (Table 6).

**Table 6:** Renal outcomes of both treatment groups.

Patients’ kidney function	Baseline		Study exit		AMD^£^ (95% CI)	P Value
	Control	Intervention	Control	Intervention		
	(n = 98)	(n = 94)	(n = 98)	(n = 94)		
**eGFR (mL/min/1.73m**^**2**^)	88.5 ± 26.1	90.4 ± 21.4	85.5 ± 23.2	90.8 ± 19.8	3.93 (1.27–6.58)	<0.01^*^
**Creatinine (mg/dL)**	70.6 ± 24.5	75.5 ± 24.9	71.1 ± 27.1	72.3 ± 25.5	−3.22 (−5.74 – −0.69)	<0.05^*^
**Albumin (g/dL)**	40.2 ± 11.1	36.6 ± 8.9	38.3 ± 5.9	36.9 ± 4.4	2.35 (−0.31–5.01)	0.08
**Albumin/creatinine ratio (mg/g)**	15.2 ± 53.1	14.3 ± 35.2	16.5 ± 57.9	13.1 ± 32.3	−2.78 (−3.98 – −1.59)	<0.01^*^
**eGFR below 60**	13 (13.3)	12 (12.8)	17 (17.3)	11 (11.7)	–	<0.01^¥^

Data are presented as Mean ± SD or number (%); AMD, Adjusted mean difference; eGFR, Estimated glomerular filtration rate.

^¥^Pearson Chi-Square test.

^£^Adjusted mean difference between intervention and control groups’ mean difference.

*Significant P value <0.05.

Moreover, in the intervention group, the mean albumin/creatine ratio was decreased to 13.1 ± 32.3 mg/g at the end of the study compared to 14.3 ± 35.2 mg/g at baseline and increased in the control group at study exit compared to baseline data (16.5 ± 57.9 vs 15.2 ± 53.1, respectively). The adjusted mean difference for the difference at study exit between the groups was significant (−2.78, 95% CI: −3.98 – −1.59, P < 0.01).

Furthermore, the prevalence of DKD (eGFR < 60, with or without high serum albumin) decreased in the intervention group compared to baseline, but not in the control group (11.7% vs 12.8% and 17.3% vs 13.3%, respectively), with significant chi-square statistics between both groups at study exit (P value < 0.01) (Table 6).

**Fig 2:**
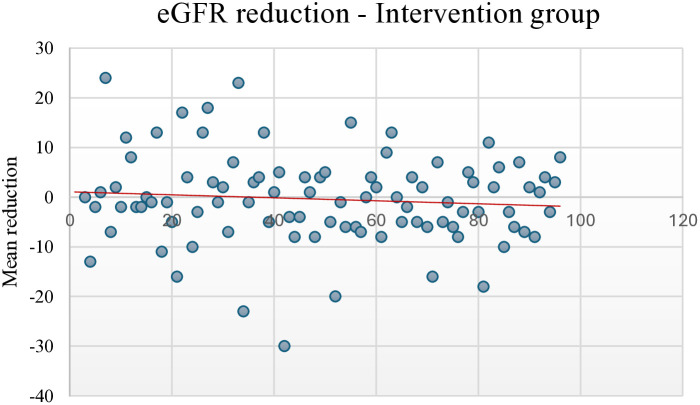
eGFR reduction in the intervention group at study exit compared to baseline. eGFR, Estimated glomerular filtration rate.

**Fig 3:**
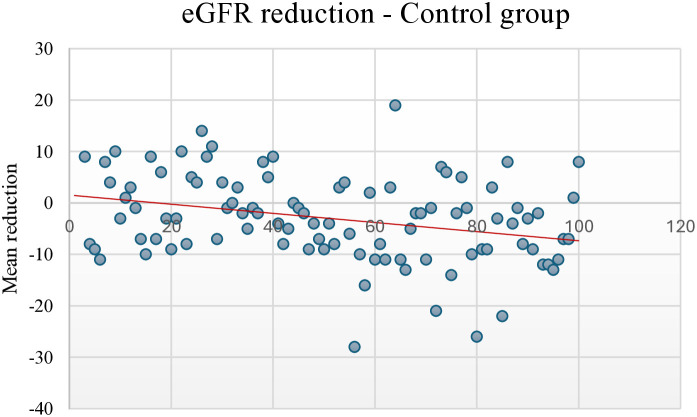
eGFR reduction in the control group at study exit compared to baseline. eGFR, Estimated glomerular filtration rate.

**Fig 4:**
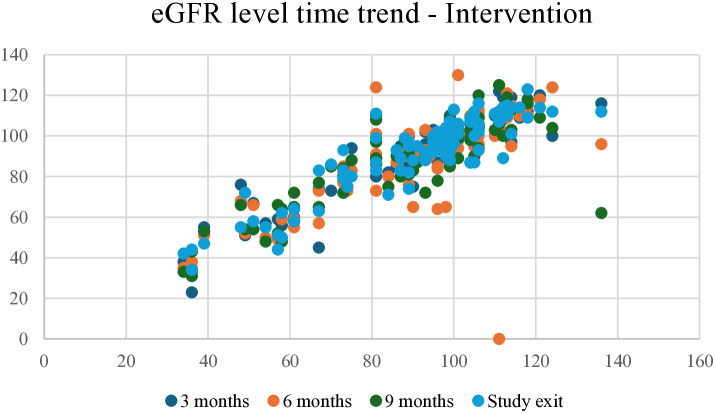
eGFR level time trend for intervention group at 3, 6, 9, and study exit. eGFR, Estimated glomerular filtration rate.

**Fig 5:**
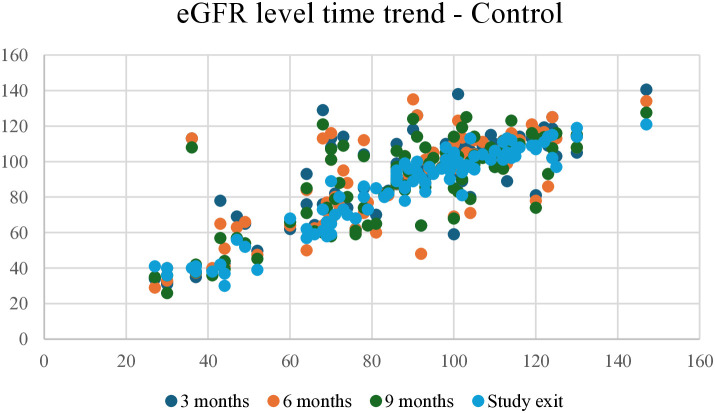
eGFR level time trend for control group at 3, 6, 9, and study exit. eGFR, Estimated glomerular filtration rate.

At the end of the study, the trend for eGFR reduction in the intervention group showed a slightly negative relationship, with fewer participants showing an eGFR reduction of >10 points. In contrast, the control group showed a stronger negative relationship, with more participants showing a reduction of ≥10 points (Figs 2 and 3). The eGFR levels over time for the intervention and control groups during the 3, 6, and 9 months of follow-up and study exit are shown in Figs 4 and 5.

#### Safety and adverse events.

A total of five medication side effect events have been reported by participants during the study, including two and three events in the intervention and control groups, respectively (Table 7).

**Table 7:** Safety and adverse events of the intervention and control groups.

Safety and adverse events	Control (n = 98)		Intervention (n = 94)		P-Value
	Base line	Study exit	Base line	Study exit	
**Medication side effects** ^**¥**^	–	3 (3.1)	–	2 (2.1)	0.68
**Cardiovascular disease**	2 (2)	4 (4.1)	4 (4.2)	5 (5.3)	0.67

Data are presented as numbers (%).

^¥^Patient-self-reported side effects.

The proportion of participants who experienced new cardiovascular events was similar in the intervention and control groups (5.3% vs. 4.2, respectively), but no difference was observed in the overall rates of hospital admission between the groups (Table 7).

## Discussion

After a mean of 11.9 months of multifactorial intervention with medication and lifestyle optimization, there was an improvement in HbA1c, SBP, and LDL-C levels among patients with type 2 diabetes who received multifactorial intervention, as compared with those who received routine care in an ambulatory care setting. In the intervention group, significant improvements were observed in HbA1c levels, from 7.4% at baseline to 7.2%; and LDL-C levels, from 3 mmol/L at baseline to 2.7 mmol/L, in the intervention group. Moreover, systolic and diastolic blood pressure have decreased at the end of the follow-up period (from 136.2 to 133.8 and 85.6 to 84.7, respectively) compared to less notable improvements in individuals who received conventional therapy. A similar outcome was reported in patients with metabolic dysfunction-associated fatty liver disease who benefited from similar combined intervention therapies, such as polypills or fixed-dose combinations, in terms of improved cardiovascular outcomes [68].

In this study, a multifactorial approach was implemented including dietary Na restriction, K or Mg supplements, K- and Mg-rich diets through dietitian counselling, and MTM to optimise electrolyte imbalance in the intervention group. The mean difference in Na levels between the groups was significant at study exit and SBP was more controlled in intervention group thanas compared to the control group at study exit. Comparable results involving Na-restricted diet have been reported in patients with type 2 diabetes and hypertension or high-normal blood pressure [69], however, inconsistent inverse associations have been demonstrated between low Na intake and mortality and increased myocardial infarction rates [70].

Although serum K levels were in the normal range at enrolment and study exit in both treatment groups, K levels were improved in the intervention group compared to the control group, highlighting the importance of the role of a dietitian in optimising dietary K with a regular follow-up diet plan [71]. The lower Na levels and greater K levels in the treatment group may have contributed to the intervention group’s reduced SBP at the end of the trial, since clinical trials have linked high Na intake and short-term K depletion to central haemodynamic abnormalities [72], leading to a significant increase in hypertension in participants with normotension or hypertension and an increase in cardiovascular mortality from any cause [73].

In the present study, both groups had mild hypomagnesemia at study enrolment (Mg level = 0.79 mmol/L and 0.8 mmol/L in the control and intervention groups, respectively). By the end of the follow-up period, the reduction of the HbA1c level from 7.4% to 7.2% in the intervention group was accompanied with an increase in the Mg level from 0.8 mmol/L to 0.84 mmol/L following the implementation of the multifactorial interventions. In contrast, Mg levels in the control group decreased from 0.79 mmol/L at baseline to 0.78 mmol/L, while their HbA1c levels increased from 7.6% to 7.8% at the end of the study. Ma et al. have repeatedly confirmed the importance of Mg in glucose metabolism, including glucose-stimulated insulin secretion, insulin action, and glucose disposal [74].

During the follow-up period, a declining trend of serum albumin levels was observed in the intervention group (from 39.9 g/L at baseline to 37.9 g/L at study exit), but not in the control group, with a slight negative trend in eGFR reduction in the multifactorial intervention group compared to a stronger negative trend in the conventional therapy group, which may be attributed to the improved glycaemic control and SBP during the follow-up period. Similar combined treatment therapies, including intensified multifactorial intervention with tight glucose regulation, have led to a reduction in the risk of both cardiovascular disease and microvascular complications [75].

Even though the proportion of participants who experienced new cardiovascular events was similar in the intervention and control groups, the cardiovascular risk factors, overall safety outcomes, and the proportion of patients with impaired kidney function have improved in the group receiving these multifactorial therapies, which include frequent dietary and exercise counselling by specialised dietitian [64], and medication adherence enforcement through an MTM program [66]. However, the implementation of such interventions in a larger clinical setting and over an extended period of time is required. Moreover, a larger patient population is needed to explore the outcomes of such multifactorial intervention with targeted counselling on Na, K, and Mg levels optimisation on cardiovascular outcomes and DKD progression.

However, this study has a few limitations in terms of the short-term follow-up period of participants (one year) because diabetes interventions usually require a prolonged time to demonstrate benefit. Additionally, the current design of our study may not effectively reduce cardiovascular diseases or progression to DKD when applied at the population level.

## Conclusion

In patients with type 2 diabetes, multifactorial intervention by a multidisciplinary team with electrolyte optimisation, diet, and exercise counselling, and comprehensive MTM have improved cardiometabolic outcomes in the study participants, including HbA1c levels, SBP, and eGFR. Additionally, it has a positive impact on cardiovascular risk factors with fewer diabetes-related medications used in Emirati patients in ambulatory healthcare clinics. However, the sustained and long-term beneficial of such interventions require a longer follow-up period and implementing them in the current therapy standards remains a major challenge.

## Supporting information

S1 File CONSORT Checklist. (DOCX)

S2 File Study Protocol. (DOCX)
