# Supplementary material for: Impact of multifactorial interventions with medication and lifestyle optimization on patients with type 2 diabetes: A randomised controlled trial
Source: PLoS One. 2025 Jul 9;20(7):e0327211. doi: 10.1371/journal.pone.0327211 (PMC12240390; doi:10.1371/journal.pone.0327211)
Supplement: S2 File — (DOCX) [file pone.0327211.s002.docx]

**Study Protocol: “Impact of Multifactorial Interventions with Medication and Lifestyle Optimization on Patients with Type 2 Diabetes: A Randomised Controlled Trial.”**

# 1. Introduction

## 1.1 Background

The prevalence of type 2 diabetes mellitus (T2DM) is increasing worldwide, with the greatest increase occurring in low- and middle-income countries [1] . The prevalence of diabetes is the highest in the Middle East and North Africa, with approximately 10.9% of the adult population being diagnosed with T2DM [2]. The estimated number of deaths caused by diabetes and its complications reached 368,000 in 2013 in the Middle East and North Africa [3]. In particular, diabetic kidney disease (DKD) is the leading cause of end-stage kidney disease and substantially contributes to cardiovascular events [4].

New approaches of managing patients with diabetes, such as pharmacist-led medication therapy management (MTM) [5], the use of telemedicine technology through mobile applications [6], dietary and exercise interventions [7, 8], and intensive serum electrolyte panel monitoring [9] have been shown to be beneficial for monitoring and managing diabetes complications, including DKD progression. However, the impact of each intervention or a combination of such interventions has not been clearly elucidated.

Although there is evidence demonstrating the benefit of individual interventions for reducing the burden of chronic T2DM and DKD, the most effective multi-factorial intervention, particularly on UAE population, remains unclear. Such an intervention is likely to involve glycaemic control by promoting adherence to medication and lifestyle, such as diet and exercise and closely monitoring to blood electrolyte levels. These factors have a long-term effect on DKD progression and hard end points, such as cardiovascular events and all-cause mortality [10].

In this study, we will examine the effect of these interventions in a combined multifactorial approach called Pharmacy Intervention Protocol (MPIP), and we will use the Abu Dhabi Health Services Company (SEHA) mobile application, which enables the patient to have access to his medical record, medications, and to book future appointments. The impact and usefulness of the application will be examined in the new pharmacy intervention protocol. Other studies have investigated similar tools in the treatment of patients with complex diseases, who require more frequent visits to achieve tighter glycaemic control or face difficulties in accessing the healthcare system [11].

Moreover, we will include a diet intervention involving pre- and post-diet assessments as well as physical activity assessments from the validated revised Summary of Diabetes Self-Care Activities measure, wherein a healthy diet plan with exercise counseling will be provided by a specialist dietician with the goal of optimising electrolyte levels, which have been demonstrated to be important in diabetes clinical outcomes, chronic kidney disease therapy and prevent progression to DKD [12]. However, more studies are warranted to prove causality.

Furthermore, we will use a stepwise approach to correct electrolyte imbalance which includes sodium, potassium and magnesium based on laboratory results, with diet as well as medication therapy modification to treat the patient’s electrolyte abnormalities. This approach has been utilized successfully in another case study to treat a patient who previously required intravenous replacement therapy for 16 years to treat electrolyte abnormalities [13].

Therefore, this randomized control trial will aim to investigate the effect of a multi-factorial intervention approach, involving pharmacist-led medication therapy management (MTM) with medication adherence, diet and exercise counselling, and optimisation of serum electrolyte levels on patients’ clinical outcomes, as well as kidney function in UAE national population with type 2 diabetes mellitus in ambulatory clinic.

## 1.2 Statement of The Problem

Up to our knowledge, this is the only research that will examine a new dimensions of multifactorial intervention approach to the local population of UAE. Limited studies investigate a multiapproach of pharmacist-led interventions in one research location to evaluate long-term adherence to medication and provide long term monitoring of eGFR to ensure that the patients are not receiving contraindicated or inappropriately high dose of medication as their eGFR declines in the progression of diabetic kidney. In addition, this research will try to explore novel solutions adapted to UAE culture to enhance adherence to dietary and exercise for type 2 diabetic patients with the aid of specialised dietitian.

Moreover, limited studies were conducted recently to investigate the effectiveness of correcting electrolyte imbalances, including serum hyponatremia, hypokalaemia, and hypomagnesemia in the progression of DKD. Few studies showed that such intervention may improve patients’ symptoms during the recovery from acute renal failure [14]. The encouraging results of the mentioned investigation have, consequently, provoked the interest in optimizing serum sodium, potassium, and magnesium to delay the progression of chronic kidney disease. Authors from several studies suggested potential avenues of future research to include investigating the effect of treating hyponatremia, hypokalaemia, and hypomagnesemia on the progression of diabetic kidney disease.

## 1.3 Research Aim, Objectives, and Outcomes

The study explores the possible interventions to improve patients’ therapeutic outcomes by medication adherence, structured diet, and exercise education. The research will investigate the impact of multifactorial interventions by pharmacists and clinical dietitian and electrolyte levels optimisation by physicians on the clinical outcomes in Emirati patients with type 2 diabetes.

The primary outcomes are as follows: (1) to evaluate the impact of pharmacist-led MTM on medications adherence, using a new pharmacist intervention protocol (MPIP), (2) to assess the effect of structure diet and exercise counseling with a specialized dietitian on anthropometry readings and carbohydrate as well as daily energy intake levels compared to control group, and (3) to assess the effect of the multi-factorial interventions on diabetic outcomes, including glycated haemoglobin A1c (HbA1c) level, blood pressure, and lipid profile.

The secondary outcomes are: (1) compare the effect of MPIP on regimen-specific medication possession ratio (MPR), and clinical interventions between both groups, (2) evaluate type, duration, and mean metabolic equivalent of task (METs) of physical activity per week during the follow up period between both groups, and (3) analyse serum electrolyte levels, cardiovascular risk factors, and eGFR levels in both group at study exit.

## 1.4 Hypothesis and Significance of The Research

In this research, we hypothesise that adherence to medication and exercise, in type 2 diabetic patients, will lead to improvement in patients’ clinical outcomes. The interventions in this research will offer recommendations for healthcare providers to adopt reliable measurable factors to improve diabetic outcomes, which eventually will lessen the burden of diabetes, medication cost in UAE and improve patient satisfaction by reducing or delaying macrovascular complications from diabetes.

In this study medication adherence will be measured by a validated Arabic version of medication adherence questionnaire, which is new in our region and in UAE. The revised Summary of Diabetes Self-Care Activities measure (SDSCA) scale will be used to evaluate adherence to diet and exercise was validated numerically. Moreover, the effects of different serum electrolytes levels including serum sodium, potassium, and magnesium levels on kidney function during the diabetes course will be investigated in this research.

The expected outcomes of the research are summarized as follow:

- Improve medication adherence measured by fixed medication possession ratio (MPR) equation and a validated questionnaire with the new pharmacy intervention protocol in the intervention compared to the control group.
- Achieve better exercise and diet adherence with overall reduction in total carbohydrate and energy intake in the intervention compared to the control group.
- Increase the number of patients reaching the target HbA1c level of < 7% and blood pressure target of 130/80 after one year in the intervention compared to the control group.
- Improved serum electrolyte levels (including serum Na, K, and Mg) in the intervention compared to the control group.
- Reduction in cardiovascular risk factors (such as wight and BMI) and improvement in eGFR levels in the intervention compared to the control group, within the study observation period.

## 1.5 Literature Review

The clinical consequence of diabetic complications and DKD are causing the most cost burden on any healthcare system. For example, the Australian commonwealth government suggest that if current trends on diabetic complications continue, spending for instant on the treatment for diabetic kidney disease will increase to $9.2 billion over the next 30 years. [15].

Many studies have investigated the correlation between pharmacist-led interventions through medication counselling in improving clinical outcomes and medication adherence in patients with T2DM [16, 17]. Moreover, the use of technology and telemedicine in disease management has been proven to be beneﬁcial in patients with diabetes and other chronic disease, demonstrating notable reduction in mortality rates [18]. Besides, the use of mobile applications can save time and effort and enable patients to manage their health more effectively [19].

In addition, several meta-analyses concluded that using carbohydrate-restricted diets in patients with type 2 diabetes resulted in better glycaemic control than control diets, they also reported different degrees of HbA1c reduction and weight loss, justified by the various levels of carbohydrate restriction in each study, and highlighted that these results are often impacted by factors other than low-carbohydrate content alone [20-23]. Moreover, several studies investigating low-carbohydrate diet interventions combined increased physical activity, and the total reduction in calorie intake with improved outcomes [24, 25].

Although several disease management models are available and supported by many healthcare systems to manage chronic diseases, such as chronic care models, and disease or case management programs to reduce long-term complications, disease cost, and overall disease burden [26], health systems do not support structured diet and exercise programs to help patients to better control their diet as well as lifestyle and improve quality of life, and focus on a self-care management approach under the guidance of family physicians or endocrinologists rather than a team approach that includes dietitians, diabetic educators, and pharmacists as part of diabetes care delivery systems [27].

Moreover, malnutrition and electrolyte imbalance are common in patients with diabetes who have chronic kidney disease and occur in varying degrees depending on the level/degree of kidney function [28]. Regular assessment of nutritional status using different measurements, proper diet history and counselling have been recommended by several existing guidelines as no single assessment can accurately determine the presence of malnutrition [12, 29].

Electrolyte disorders, which also coexist in several medical conditions, including hypertension and congestive heart failure [30, 31], were previously observed by Shahid and Mahboob during DKD progression in patients with diabetes compared with non-diabetic or normotensive patients [32]. This study showed a significant increase in erythrocyte intracellular sodium levels and a significant decrease in erythrocyte intracellular potassium, serum sodium and serum magnesium levels in patients with diabetes mellitus and nephropathy compared with those in euglycemic patients. Additionally, the attenuation of electrolyte abnormalities in patients with diabetes can reverse hyperglycaemia-induced effects on cellular transport processes and decrease NaK-ATPase function, suggesting that serum electrolytes, such as potassium and magnesium, can be considered indicators of DKD progression and play an important role in its treatment [33].

For instance, hypomagnesaemia may impair glucose excretion and play a role in the pathogenesis of some of the complications of diabetes [34]; however, further studies are needed. In contrast, hypermagnesemia in patients with diabetes was more frequent during renal insufficiency and end-stage renal disease [35, 36], and limited studies have reported on the effect of untreated hypermagnesemia on the progression of kidney disease.

Furthermore, hypokalaemia or hyperkalaemia are often observed in patients receiving healthcare [37] and may lead to several renal function abnormalities [38], which may be amplified in patients with diabetes.

# 2. Materials and Methods

## 2.1 Study Setting

The research conducted at the endocrinology and chronic disease (CDC) clinics at Oud Al-Touba Diagnostic and Screening clinic, one of the ambulatory healthcare centres in the emirates of Abu Dhabi in United Arab Emirate. Oud Altouba Clinic is part of the Ambulatory Healthcare Services (AHS) and integrated with Abu Dhabi Healthcare Company (SEHA), the largest healthcare network in UAE.

The AHS clinics provide the most comprehensive health care services with more than 43 speciality services, including Chronic Disease Clinic (CDC), endocrinology, cardiology, family medicine, and nutrition clinics. Besides, a pharmacy providing specialised clinical services, and patient and family health education programs.

## 2.2 Research Design

This is a randomised controlled, multidisciplinary trial. This research is a single-centre study, involving a 12-month follow-up period or till the date of participant withdrawal due to any cause, whichever comes first. The Study is not funded or supported by any grant or institute.

## 2.3 Study Participants

The study will recruit Emirati patients previously diagnosed with T2DM from the endocrinology or chronic disease (CDC) clinics, who have will match the research inclusion criteria and have been taking the same diabetes medications for 12 weeks before the study with regular follow-up schedules in the previous year. Eligible patients will be randomized and blinded to the intervention and control groups.

The patients will be included in the analysis are those whose records are available in Salamtak (electronic medical record system operated by the Salamtak platform). At the end of the study, those with a medical history of cancer, chronic liver disease, high cardiovascular risk (defined as a 10-year predicted atherosclerotic cardiovascular disease (ASCVD) risk of ≥7.5% using the pooled cohort equation or a Framingham Risk Score of ≥20%), cardiac surgery within the last 12 months or any diabetic macrovascular complications will be excluded. Moreover, patients will be excluded either if they withdraw from the study or have a missed follow-up data including missed laboratory tests at the end of study period as per study protocol.

## 2.4 Inclusion Criteria

- Male or female Emirati patients between 30 and 65 years of age.
- The patient has been previously diagnosed with type 2 diabetes mellitus recorded in Salamtak, defined in our diagnostic criteria.
- Previously on diabetic medication recorded in Salamtak and antihyperglycemic therapy must be unchanged for 12 weeks before the study.
- Patients with normal or mildly/moderately abnormal serum magnesium and/or potassium levels.
- Patients with normal to moderately impaired renal function, defined as an estimated glomerular filtration rate (eGFR) of >30 mL/min/1.73 m2, for more than 3 months at baseline (stages G1, G2, G3a and G3b of chronic kidney disease) [39].
- Body Mass index less than 40 at study enrollment.

## 2.5 Exclusion Criteria

- Non-Emirati patients or patients without full insurance coverage.
- Patients with severe or symptomatic hypo- or hypermagnesemia and/or hypo- or hyperkalaemia, metabolic acidosis or hypophosphatemia with or without proximal renal tubular acidosis and Fanconi syndrome.
- Patients with prolonged hypokalaemia with surreptitious diuretic use, laxative abuse, eating disorders or primary aldosteronism.
- Patients receiving medications that may cause drug-induced acute renal failure during the observational period and may be implicated in hypomagnesaemia (e.g. aminoglycoside antibiotics, cyclosporine, amphotericin B, cisplatin, pentamidine and foscarnet).
- Patients who have undergone bariatric surgery within the past two years or other gastrointestinal surgeries that induce chronic malabsorption.
- Pre-menopausal women who are nursing or were pregnant within the last 12 months.
- Blood dyscrasias or any disorders causing haemolysis or unstable red blood cell population (e.g., malaria, babesiosis, haemolytic anaemia).
- Stage 4 or 5 CKD patients, with a severe reduction in glomerular filtration rate, kidney failure or dialysis, defined as GFR ≤ 29 ml/min or albumin/creatinine ratio ˃ 30 mg/mmol as baseline value.
- High cardiovascular risk, defined as 10-year predicted atherosclerotic cardiovascular disease (ASCVD) risk ≥7.5% by Pooled Cohort Equation or Framingham risk score ≥20%, or cardiac surgery, or angioplasty within the last 12 months or any diabetic macrovascular complications as defined in our definition.
- Indication of liver disease, defined by serum levels of either alanine aminotransferase (ALT), aspartate aminotransferase (AST) or alkaline phosphatase (ALP) above 3 x upper limit of normal as defined at the beginning of the study or within the last 12 months.
- Medical history of cancer and/or treatment for cancer within the last five years, or immune compromised patients.
- Treatment with systemic steroids or change in dosage of thyroid hormones within the last 12 months after starting the study or any other uncontrolled endocrine disorder.
- Patient with communication barriers that may affect obtaining patient adherence, receiving diet, exercise counselling or consent signing, and include:
- a. Patients with severe emotional distress.
- b. Patients who are unable to use mobile applications or access the internet for any reason.

## 2.6 Randomization

Randomization processes will be performed by a computer-generated random number in Excel Microsoft Office to generate an allocation sequence for each participant in the intervention and control group by the equation (=UNIQUE(RANDARRAY(239,1,1,300,FALSE)), with allocation concealment by sequential numbering representing the participant’s turn in joining the study. All patients identifying data will be removed to conceal randomisation and minimise predictability of the generated random sequence.

Each patient will be assigned a code documented in the consent form and the data entry sheet by the principal investigator, and this information will be saved in a password-protected file. Participants in the intervention group will be identified electronically one week before the next refill schedule or follow-up visit. The control group will continue to receive routine care provided in the clinic during the study period without any interfere in their follow-up appointments.

## 2.7 Patient Consent

At the physician’s office, the doctor will discuss the possible participation of the eligible patient in the study, and if satisfied, the participant will sign the consent form (Appendix 1) and receive a copy of it along with the study information sheet (Appendix 2).

The trial will be submitted and monitored by SEHA Research Ethics Committee (REC) after approval. The results will be communicated to the SEHA REC in the terminal report and to the public in a peer-reviewed journals. Moreover, the physicians from the Oud Al-Touba clinic will have access to the clinical trial protocol and research results, in a way that protects patient’s confidentiality, to advance medical research and understanding, and any modifications in study protocol (if needed) will be submitted to SEHA REC for approval.

Patients’ confidentiality will be maintained during the study, and all de-identified protected health information will be recorded electronically and saved securely by password protection, limiting the access to authorised research personnel only and SEHA REC for auditing and monitoring purposes during the study period.

Authorship agreement to declare the intellectual contributions of the research team, origin of the research and accountability of any published information was written and will be reviewed by the research team before publication.

The research is not sponsored by any company, and the researchers have declared that they have no known notable financial interests that would reasonably appear to affect the research. Although participants will not be compensated for their extra time spent during interviews or for any inconvenience that may result from taking extra medications or blood tests every 3 months, they will grant prioritised access to physician’s appointments, including dietician’s visits. Furthermore, they will grant direct access to our cardiologist in case of any harm from prescribing medicines or abnormal laboratory results, without any additional cost. Moreover, a 12-h counselling hotline will be available during and after the study follow-up period to answer any queries or report any side effects.

## 2.8 Study Procedures

Eligible participants will sign the consent form at the physician’s office, and laboratory results will be recorded as baseline data. If the patient’s medical record does not contain recently updated laboratory tests from the previous three months, laboratory tests will be scheduled for blood sample collection in the clinic and tested in Abu Dhabi Health Services Company (SEHA) central laboratory, and the same process will be performed at the conclusion of the follow-up period after 12 months. Laboratory tests in the study protocol included gylcated hemoglobin A1c (HbA1c), estimated Glomerular Filtration Rate (eGFR), complete blood count, electrolyte panel including potassium and magnesium levels, serum creatinine levels, and albumin/creatinine ratio.

Laboratory and clinical assessments in the study protocol included BP measurement and laboratory tests HbA1c level, low-density lipoprotein cholesterol (LDL-C) level, complete blood count, electrolyte panel including K and Mg levels, eGFR, serum creatinine level, and albumin/creatinine ratio. BP measurements and laboratory tests will be performed according to the standard procedures for BP measurement and blood sample withdrawal at the clinic.

In addition, each participant in both groups will undergo individual assessments at initial visit and study exit including medication adherence by clinical pharmacist and diet and exercise assessment by specialized dietician.

During the study period, the control group will receive the usual care provided in the clinic including the routine follow-up and clinical assessments (as needed). The routine usual care will include follow-up schedule every 3 months with the treating physician, vital signs monitoring in the visited clinic, physician consultation, any additional investigational services or laboratory tests requested by the treating physician depending on each patient’s condition, patient counselling at the pharmacy counter, dietitian counselling service if requested by the patient or referred by the treating physician, and a call reminder from the clinic call centre 3 days before the appointment.

Moreover, the control group will have their HbA1c levels recorded at least twice yearly (at initial and exit visits) in patients with stable glycaemic control meeting the treatment goals and quarterly in patients not meeting the treatment goals, which will be at the discretion of the attending physician [40]. Other laboratory tests will be performed twice yearly (or more frequently as per the physician’s request), and any major adverse cardiac events, emergency room visits or hospitalisations will be recorded and compared with baseline data.

The intervention group will receive multi-factorial interventions and assessments at initial visit and at each follow-up visit every 3 months after enrolment in the study. The multi-factorial interventions composed of the three below approaches: medication adherence counselling with medication therapy management program, diet and exercise counseling, and serum sodium, potassium and magnesium optimization (Figure 1).

- Adherence and MTM assessment.
- Diet and exercise assessment.
- Clinical outcomes and electrolyte level assessment.

Close-out

12-month visit

12-month visit

- Adherence counselling and MTM.
- Diet and exercise intervention.
- Multifactorial interventions with electrolytes level optimization.

Interventions

6-month visit

3-month visit

1-month visit

9-month visit

- Baseline laboratory tests, Na/K/Mg levels .
- Medication adherence questionnaire.
- Diet and exercise assessment.

Pre-assessment

Initial visit

Initial visit

Eligibility – Inclusion - Randomisation (1:1)

**Intervention Group**

**Control Group**

Enrollment

Figure 1: One-year, randomised control study.

FMPR= Fixed medication possession ratio, K = Potassium, Mg = Magnesium, MTM = Medication therapy management.

### 2.8.1 Medication Adherence and Medication Therapy Management (MTM)

We will examine a multi-factorial pharmacy-led interventions protocol (MPIP) that aims at maximizing patients’ adherence. Patients in the intervention group will receive comprehensive patient counselling designed to improve participant understanding and enhance appropriate use of medications, with a follow-up phone interview within 2 weeks after each follow-up visit. Moreover, we will examine medication therapy management (MTM) intervention as distinct group of services provided to each participant in the intervention group as part of MPIP and occur in conjunction with medication adherence counseling.

MTM encompasses of several activities including performing comprehensive medication reconciliation and review to identify as well as resolve any medication related issues or adverse events, modifying or optimizing medication therapy in coordination with health care providers, monitoring, evaluating or requesting necessary laboratory and assessments of participants health status, and finally formulating a pharmaceutical care plan to evaluate and monitor patient’s compliance and response to therapy. In addition to other support services such as medication booklet and mobile application to optimize patient clinical outcomes to their therapeutic regimens.

Intervention procedure:

At baseline, participants in both groups will receive medication counselling by a clinical pharmacist as part of the usual care provided in the clinic, and baseline assessment of medication adherence will be performed using a validated medication adherence questionnaire (Appendix 3) [41]. Participants will be instructed to report any possible side effects or factors that may affect their medication adherence, and to reveal any modifications in lifestyle activities or eating habits during the study.

Participants in the intervention group will join the MPIP program on their first visit and will receive two multifactorial interventions: medication adherence counselling and medication therapy management, as detailed below.

1. Medication Adherence Counselling

Each participant in the intervention group will receive a 15- to 30-min counselling session at the initial visit and each scheduled follow-up visit which will occur every 3 months. During these sessions, the pharmacist will assess medication adherence with a validated adherence questionnaire (Appendix 3) and counsel the patients on the importance of medication adherence on clinical outcomes. The barriers to medication adherence will be identified, discussed with the patient, and resolved as much as possible. Finally, the patient will be provided with two or three months of medication supply, and the medication refill date will be documented for medication adherence calculation using the medication possession ratio (MPR) [42, 43].

After each visit, each participant in the intervention group will receive a 15-minute follow-up phone interview within two weeks for adherence reinforcement and to address any non-compliance issues or medication side effects. Moreover, they will be scheduled for their next medication refill and follow-up visits in the clinic appointment system. Appointments will be scheduled at months 3, 6, and 12 from the initial visit according to the patient’s preferred date, and they will receive a reminder phone call three days before the next visit from the clinic call centre.

Medication adherence will be evaluated using two techniques during the follow-up period: calculating the medication adherence possession ratio and the medication adherence questionnaire scores.

1. Medication Adherence Possession Formulas

In this study, we will use three formulas to calculate medication adherence from patients’ medication refills recorded in Salamtak:

1. Total Fixed Medication Possession Ratio (MPRt)

A fixed medication possession ratio (MPR) formula will be used to measure total (composite) adherence to all medications (MPRt) [44, 45]. The MPRt will be calculated for three chronic regimens including antihyperglycaemic, antihypertensive and antihyperlipidaemic regimens.

For the total MPRt, no regimen-specific MPR (MPRs) will be measured. It is calculated by summing the days’ supply of medication per prescription, then dividing by the number of days of patient follow-up, using a fixed follow-up period of 365 days (12 months) as a constant denominator, adjusted to hospital stay if the participant was admitted during the follow-up period (Figure 2).

$$\text{MPR}\text{t}\text{ }\text{=}\frac{\sum_{i = 0}^{End of follow-up period} \text{Days of supply Rxi}}{\text{Days of follow-up period}}$$


$\text{MPR}\text{t}\text{ }\text{=}$

Figure 2: Fixed total medication possession ratio (MPRt) calculation

For the above example, if the patient had a medication supply equal to 345 days (90 days + 75 days) during the fixed follow-up period of 365 days, the MPRt calculated as 345/365 = 0.95.

1. Regimen-specific Medication Possession Ratio (MPRs)

Regimen-specific MPR (MPRs) will be calculated for all three regimens (antihyperglycaemic, antihypertensive, and antihyperlipidaemic), whether any or all were contained in the patient’s medication profile [46, 47].

The denominator for MPRs also covers the follow-up period of 12 months but will be calculated based on the last refill date for a specific medication regimen (for any medication class) minus the first refill date for any medication in the regimen, even if this medication was in a different class from the medication used in the last refill. In addition, medications within the same regimen will be considered interchangeable (even if they belonged to different medication classes).

$$\text{MPRs}\text{ }\text{=}\frac{\sum\text{Days of supply of }\text{medication}\text{ in a regimen}}{\text{Last refill date‐First refill date} \left( \text{for specific regimen} \right)}$$

Of note, for both types of MPR (MPRt and regimen-specific MPRs), if the patient had refilled medication prior to the date of joining the study, MPR will be calculated from the date of signing the consent, excluding the supply from the last prescription or medication refill and will be capped at 1, and medication adherence (either MPRt or MPRs ) will be calculated after 12 months of follow-up [42].

The standard adherence threshold of 0.80 MPR will be used as a lower bound for mediation adherence [45], with MPR = 1 indicating perfect adherence, MPR < 0.80 indicating patient non-adherence, and MPR= 0 indicating no adherence. The patients’ medication refills for each medication class will be recorded from Salamtak (including records from private and governmental pharmacies) at each follow-up visit.

1. Regimen Non-persistence Possession Ratio (RNP)

In addition to the MPR calculation, we will estimate the regimen non-persistence (RNP) or days without medication for each regimen (diabetic, hypertensive, and antihyperlipidaemic regimens) the patient had during the study [48], calculated as the total number of days the patient did not receive one or more medications for any of the patient’s chronic regimen divided by the total follow-up days.

$$\text{RNP}\text{ }\text{=}\frac{\sum\text{Days of supply of medication in a regimen}}{\text{Days of follow-up period}}$$

1. Medication Adherence Questionnaire

A validated medication adherence questionnaire translated into Arabic, showing sufficient reliability with adequate internal consistency, will be used initially to measure medication adherence at baseline for all study participants, and subsequently at each follow-up visit in the intervention group and again at the study exit (Appendix 3)[41].

The questionnaire contains four medication adherence questions from the validated Arabic version of the tool: 1) “Do you ever forget to take your medicine?” and 2) “Are you careless at times about taking your medicine?”, 3) “When you feel better do you sometimes stop taking your medicine??”, 4) “Sometimes if you feel worse when you take the medicine, do you stop taking it?”.

The total score of the medication adherence questionnaire included the first four questions with scores ranging from 0 and 4, with “Yes” having score = 1 and “No” = 0. Increased levels of adherence correspond with lower scores. Patient scores from 3 to 4 will be classified as low adherence, moderate adherence (score from 1 to 2), and high adherence (score = 0, with no answers with yes) [49] (Table 1).

Table 1: Medication adherence questionnaire scoring scheme

| Adherence questionnaire scores |
| --- |
| High adherence = 0 item answered “Yes” |
| Moderate adherence= 1 or 2 items answered “Yes” |
| Low adherence= 3 or 4 items answered “Yes” |

1. Medication Therapy Management (MTM) Program

As a new component of the MPIP, the MTM program will be made available to the clinic’s ambulatory service for the first time, and the intervention group will receive the following interventions as part of the program:

- 1. Medication counselling and reconciliation

MTM will be effectuated during the counselling session for 15–30 min at the time of dispensing medication. Each patient interview will implement the five criteria for MTM, including medication review to optimise patient treatment as per the latest treatment guidelines, assessment of the possibility of deprescribing, evaluation of any prescribed non-formulary drugs, patient counselling, and provision of printed medication educational materials, particularly for new, high alert, and narrow therapeutic index medications from an evidence-based drug reference to ensure the safe and proper use of these drugs (Lexicomp Online) (Appendix 4).

Moreover, the counselling session will include counselling on potential drug-drug or drug-food interactions and the proper use of any device or over-the-counter (OTC) medication the patient was using. Any detailed disease information or drug modifications required will be discussed and resolved by the treating physician.

Medication reconciliation and chart review will be carried out through the Salamtak local network, integrated with all governmental hospitals, ambulatory clinics, and private institutes in the Emirates of Abu Dhabi in the UAE.

The number and type of pharmacy clinical interventions, in addition to antihyperglycaemic deprescribing, will be analysed and compared between the two groups at the end of the follow-up period.

- 1. Patient Medication Booklet

A pocket-sized patient-specific medication booklet will be provided to each participant in the intervention group (Appendix 5). This initiative in the MPIP aims to promote medication adherence in elderly patients and those with low digital skills. Moreover, the booklet helps to track OTC drugs or herbal medications bought from community pharmacies and enables patients as well as community pharmacists to fill in the name of the drug and the dose by hand, which are then manually added to the patient’s medical record in Salamtak at the next MPIP visit.

During the counselling session, the counselling pharmacist will attach the prescribed medication labels to the booklet and will ensure that the booklet is filled with an updated list of all prescribed medications, medication doses, allergies, next scheduled refill, and any OTC drugs the patient is currently taking (Appendix 5).

Patients will be advised to bring the medication booklet to each follow-up visit to update the next refill schedule or any changes in their profiles. Moreover, it will be used as a reference during Salamtak downtime or when patients’ medical records from other private facilities are not available.

- 1. SEHA Mobile Application

Despite the fact that the SEHA mobile application [50] is accessible to all patients for download and usage, the patients often do not know how to use its features. As a new initiative in the MTM program, participants in the intervention group will receive face-to-face educational sessions for 5 minutes on the use of the SEHA mobile application, how to read the clinical information and laboratory results, extract their medication profile, add OTC medications to their medical record, and read notifications for medication approval from insurance as well as for subsequent medication refills.

The SEHA mobile application provides real-time individualised feedback, including patient access to Salamtak, laboratory test results, radiology reports, medications, allergies, immunisation records, and health maintenance history for patient visits occurring in SEHA facilities. Moreover, it enables patients to request or reschedule appointments or reschedule them, and search for a healthcare facility. The application sends a short message service (SMS) to the registered mobile number for appointments, with reminder notifications to remain keep the patient informed about future or follow-up appointments.

At the end of the study, we will use the SEHA mobile, and booklet use questionnaire to record the number of participants in each group using the application on their mobile device and the extent of application utilisation (Appendix 6). The results will be correlated with medication adherence to measure the effect of online technology on medication management.

### 2.8.2 Diet and Exercise Intervention

In addition, we will investigate the effect of structured diet therapy and exercise counselling for diabetic patients by registered dietitian, on anthropometry measurements including weight, body mass index (BMI), and waist and hip circumference. The intervention group will also receive educational adherence session at each follow-up visit by the same dietician to reinforce optimal diet and exercise for each participant to ensure balanced caloric and mineral intakes and effective lifestyle modification.

Intervention procedure:

A) Dietary and Exercise Assessment

All participants of the control and intervention groups will undergo individual pre-assessment of diet and physical activity using the revised version of the summary of diabetes self-care activities (r-SDSCA) measure questionnaire which consists of two core subscale questionnaires (i.e. dietary and physical activity subscale questionnaires (Appendices 7 and 8) to assess self-care management in patients with diabetes [51]), with adequate internal test-retest reliability and evidence of validity and sensitivity to change [52].

The questionnaire will be completed by a specialised dietitian at the initial visit and at each follow-up visit in the intervention group (or at study exit in the control group) to measure participants’ adherence to diet goals and exercise recommendations in an education session for 30 to 45 minutes.

The scale, utilise the metric “days per week” instead of using percentages to cover diabetes self-care activities and adherence, and the questions should cover the past 7 days. If the patient was sick during the past 7 days, the dietitian will ask them to recollect the last 7 days when they were not sick. Each question uses the number of days per week on a scale of 0–7.

B) Usual Care Diet and Exercise Counselling

After recruitment, study participants in both groups will receive basic diet and exercise counselling, in addition to advice on healthy eating for diabetic patients with printed materials (Appendix 9) as part of usual care in the clinic.

Moreover, both groups will complete a pre-assessment nutrition knowledge questionnaire with eight questions evaluating knowledge of the importance of frequent laboratory blood tests; regularly monitoring of kidney function; recommended dietary allowances of Na, K, and Mg; food sources of these elements; and symptoms of deficiency. Moreover, they will receive educational information based on dietician evaluation as part of routine care in the clinic (Appendices 10 and 11).

Participants in the control group will not receive any advice on carbohydrate calculation, healthy plate model, or specific exercise instructions during the study period. Participants in all groups will be asked to report any modifications in their usual diet or activities throughout the study period.

C) Structured Diet and Dietary Carbohydrate Count Education

The intervention group will receive an educational reinforcement session for a structured diet at each follow-up visit by the same specialised dietitian. Dietitian appointments will be performed concurrently after each follow-up visit to the treating physician appointment to build on physician advice; and included nutrition counselling to reinforce balanced caloric and mineral intake, as well as measuring adherence to structured diet plans.

In the structured diet therapy, the dietitian will set individualised carbohydrate (carb) targets for each participant for eating meals or snacks during the day to cover daily macronutrients, considering the participants’ current eating patterns, food preferences, motivation, and ability to follow the recommended diet.

The total daily calories required by each participant will be calculated according to the United Arab Emirates (UAE) ministry of health and prevention recommendations [53], and based on the basal metabolic rate (BMR) calculations [54] considering the appropriate activity factor based on the participants’ lifestyle and work (Table 2).

Table 2: Calorie-calculation based on BMR and activity factors.

| Lifestyle and exercise frequency | Activity factor |
| --- | --- |
| Sedentary (little or no exercise) | BMR x 1.2 |
| Lightly active (light exercise/sports 1-3 days/week) | BMR x 1.375 |
| Moderately active (moderate exercise/sports 3-5 days/week) | BMR x 1.55 |
| Very active (hard exercise/sports 6-7 days a week) | BMR x 1.725 |
| Extra active (very hard exercise/sports & physical job or 2x training) | BMR x 1.9 |

Basal metabolic rate (BMR) calculations for men and women:

BMR for men= [weight (kg) x 10] + [height(cm) x 6.25] – [age(y) x 5] + 5

BMR for women = [weight (kg) x 10] + [height(cm) x 6.25] – [age(y) x 5] – 161

The calculated amount of carbohydrates will be approximately 4–5 carb exchange for males in their main meals (approximately 60–75 g of carb), and for females 3-4 carb exchange in their main meal (approximately 45–60 g of carb), with one to two carb exchanges in their snacks. As part of the structured diet intervention, participants in the study will be instructed to maintain a daily intake of 75–105 g of carbohydrates for males and 60–90 g of carbohydrates for females, divided between the main meals and snacks based on their basal metabolic rate calculations.

Moreover, participants in the intervention group will receive educational materials on the amount of carbs and calories in food and the different methods of measuring food portions by hand, cups, or spoons and to calculate a portion size of 15 grams of carbohydrate to an equal one serving “exchange” (Appendix 12). In addition, participants will be educated on the use of a healthy plate model to prepare their meals [55]. They will also receive educational materials with photos showing the amount of carbs in bread, cereals, rice, and pasta (Appendix 13).

At each scheduled follow-up visits occurring every 3 months (≥3 scheduled visits during the follow-up period), the dietitian will support the intervention group patients in modifying their structured meal plans and achieving their goals throughout the study period. Moreover, a 15-minute phone coach session once per month will be used to motivate the patients to adhere to their structured meal plans, provide guidance on carbohydrate calculation for any additional food preferences, implement a new structured meal plan, and answer any queries concerning diet or exercise interventions.

Dietary carbohydrate value and daily energy intake will be calculated from the patient’s food log for 3 days including a weekend before the first baseline visit and at study exit for all participants, and before each follow-up visit every 3 months for the intervention group. Participants will be asked to fill the food log with all consumed food and beverages measured by hand, cups, or spoons and by portion, weight, or volume for other individual-serving snacks, foods, or beverages outside the home.

In each food log section, the participants can describe the consumed food or beverage, and any other oil, fats, sauces, or condiments used in preparation or added during food serving, in addition to the time and occasion of each meal or snack. During the session, the research dietitian will review the food logs, clarify any unclear entries or descriptions, and estimates the average daily energy intake and dietary carbohydrate value for each participant.

D) Exercise and Physical Activity Reinforcement Counseling

For each participant in the intervention group, the dietitian will develop individual exercise targets based on their clinical condition and motivation level while taking into consideration each participant’s current eating patterns and willingness to adhere to exercise and lifestyle modifications.

Any identified challenges that limit the patient from being physically active will be discussed with the patient, and an action plan will be discussed with the treating physician and documented in the patient’s file.

Anthropometric measurements will be obtained by registered nurses in the clinic at the time of enrolment, before exiting the study for all participants, and at each follow-up visit for the intervention group. Body weight and height will be measured without shoes using a standard monthly calibrated scale at the Dietitian Office (Tanita Corporation, Japan). The same nurse will measure the patients’ waist circumference just marginally above the hipbone and their hip circumference at the largest part of their buttocks.

In addition, to explore the type of most recurring physical activity in our intervention population, we added additional table to the questionnaire to describe the type of physical activity, intensity of the purposive activity, average number of days per week for each activity, and duration of each activity (Appendix 14).

Furthermore, in the absence of a gold standard to classify physical activity intensities, we will calculate the absolute intensities of the exercise sessions based on the recommendations from the American college of sports medicine and the American heart association [56], and the British Association for cardiovascular prevention and rehabilitation (BACPR) [57]. Each activity will be expressed in metabolic equivalent of task (METs) values and classified as light, moderate or vigorous intensity with Borg’s CR10 scale [58] to provide subjective rating of perceived exertion. Guidelines showed that combining both tools augment the relation between fitness levels and exercise intensity [59]. Moreover, to allow homogeneous and adequate analysis of METs values, the mean duration of the training session from patients’ interview will be considered within three categories: 30 minutes or less = 30 minutes, 31 to 60 = 60 minutes, and > 60 min = 90 minutes.

Besides, we will calculate the average metabolic equivalent of task per week based on the corresponding METs of each activity that match with CR10 intensity score (Table 3) multiply the average duration of activity performed in the number of days of activity performed during the week (For example, walking 3.0 mph with CR10 =4 (moderate), equals to 3.3 MET (code:17190) [60], for 30 min on 3 days per weeks [3.3 MET x 90 min] = 2970 MET-minutes per week).

Table 3: Exercise intensity based on CR10 modified scale.

| CR10 exercise intensity scores | MET equivalents |
| --- | --- |
| Low exercise intensity = 0, 1, or 2 | Light < 3.0 METs |
| Moderate exercise intensity = 3 or 4 | Moderate 3.0 – 6.0 METs |
| High exercise intensity = From 5 to 10 | Vigorous > 6.0 METs |

### 2.8.3 Electrolyte Optimization Including Serum Sodium, Potassium as well as Magnesium Levels

During the follow-up, the intervention group will be evaluated for electrolyte imbalance, particularly Na, K, and Mg levels. The metabolic panel (including serum Na, K, and Mg levels) and eGFR will be measured at baseline and at 3, 6, 9 and 12 months after recruitment. Moreover, participants will be evaluated for symptoms of electrolyte imbalance. In patients with no or minimal symptoms of mild to moderate hyper-, hypo-, or hypernatremia [61, 62] hypomagnesaemia [63] and/or hyper- or hypokalaemia [64, 65], treatment is initiated based on the recommended clinical pathway with the primary goal of preventing or treating life-threatening complications while diagnosing and treating the underlying disease [66-68].

At the end of the trial, we will assess the effect of serum electrolyte level optimization during medical investigation on diabetes management, clinical outcomes, as well as kidney function measured by estimated glomerular filtration rate (eGFR) levels.

## 2.9 Rules for Early Stopping

The patient had the right to withdraw consent and stop or postpone the assessment interviews or adherence questionnaires if he/she became upset at any time during the study period. The participant may choose to stop participating in the study at any time without any penalty or limitation in the usual clinical services provided. Any adverse event and/or unanticipated problem involving any risk to the participants will be reported to the SEHA REC. Moreover, any problems that may arise, such as significantly abnormal laboratory results, medication side effects or complications in blood sample collection, will be treated instantly in the urgent care unit inside the clinic or referred to the proper management pathway.

## 2.10 Statistical Analysis

The sample size required to reach significant in the three primary outcomes in the study’s multi-intervention approach is 180 participant (90 participants in each group). Sample size was calculated with Giga sample size online calculator [69], according to a pilot analysis of 50 participants matching study protocol, with a power of 80% with alpha level of 5% to detect the minimal difference of each primary outcomes in the study.

For 1^st^ outcome, the baseline medication possession ratio (MPR) for those 50 participants was 0.93 ± 0.09 (mean ± SD), we determined that a target of 82 participants per group would be required for a minimum detectable effect of 0.035 between both groups. A total sample size of 180 was calculated, assuming dropout rate of 10% in both groups.

For 2^nd^ outcome, the mean body mass index (BMI) (mean ± SD) of the 50 participants was 31.2 ± 6.4, and the total number of participants required to detect minimum detectable difference of 2.5 in BMI between groups was 180 participants, powered for 80% with α equal to 0.05, considering drop rate of 10% throughout the study period.

In addition, for 3^rd^ outcome to detect minimal difference of 0.5% in HbA1c between groups with mean HbA1c = 7.7 ± 1.3 (mean ± SD) at baseline (for the same 50 participants), a total sample size of 184 participants were calculated with 84 participants per group were required, considering 10% expected dropout rate during the follow-up period.

For data analysis, the difference between both groups will be tested for normality using the Shapiro–Wilk test. Data will be presented as percentages for categorical variables or mean ± SD or median (25th, 75th percentiles) for continuous variables. Chi-square test will be used for the categorical variables, whereas student’s t-test will be used for normally distributed continuous variables, or Mann–Whitney U test for non-normally distributed continuous variables.

Analyses will be performed using IBM SPSS Statistics (version 26), and differences will be considered significant for a p-value of <0.05.

Data verification will be performed on a weekly basis, and any missing data will be completed within 1 week of initial data collection and will be doubled-checked by two researchers before data analysis. Missing data in the final dataset will be filled by multiple imputation of the incomplete multivariate data using NORM® programme (version 2.03) integrated with SPSS, under a normal model and with two-level hierarchical linear modeling (HLM) analysis.

# 3. Participant timeline

Table 4: Participant timeline and expected starting and finishing times.

| **No.** | **Protocol Item** | **Starting Date** |
| --- | --- | --- |
| 1 | Conducting detailed literature review, mapping the research field and identifying the direction of the study. | July 2020 |
| 2 | Conducting preliminary study to identify the feasibility of laboratory tests, diet and medication counseling and follow-up strategies in the research site. | October 2020 |
| 3 | Designing the research methodology and providing detailed recruitment, randomization, and follow-up procedures. | November 2020 |
| 4 | Preparing studies materials (consent form, patient’s research summary information, printed questionnaires, and patient’s educational materials) | November 2020 |
| 5 | Submit the research protocol to AHS, SEHA ethics committee. | January 2021 |
| 6 | Receiving AHS, SEHA ethics committee and submitting it to UAEU ethics committee. | April 2021 |
| 7 | Patients screening and eligibility check | April to June 2021 |
| 8 | Patient recruitment and initial assessment starts | July 2021 |
| 9 | Patient recruitment ends | September 2021 |
| 10 | First phase of intervention initiated after 3 months from study entry. (Depending on the participant’s initial assessment and appointment schedule). | November 2021 to January 2022 |
| 11 | Second and third intervention phases at 6th and 9th months follow-up visits | February 2022 to July 2022 |
| 12 | Study ended with the last patient follow visit in the intervention group | September 2022 |
| 13 | Collecting research data, data verification, and performing quality control validation of collected data | October and December 2022 |
| 14 | Data analysis | January 2023 till April 2023 |
| 15 | Results writing and revision, exit report for ethics committee and publications | 2023 till 2024 |


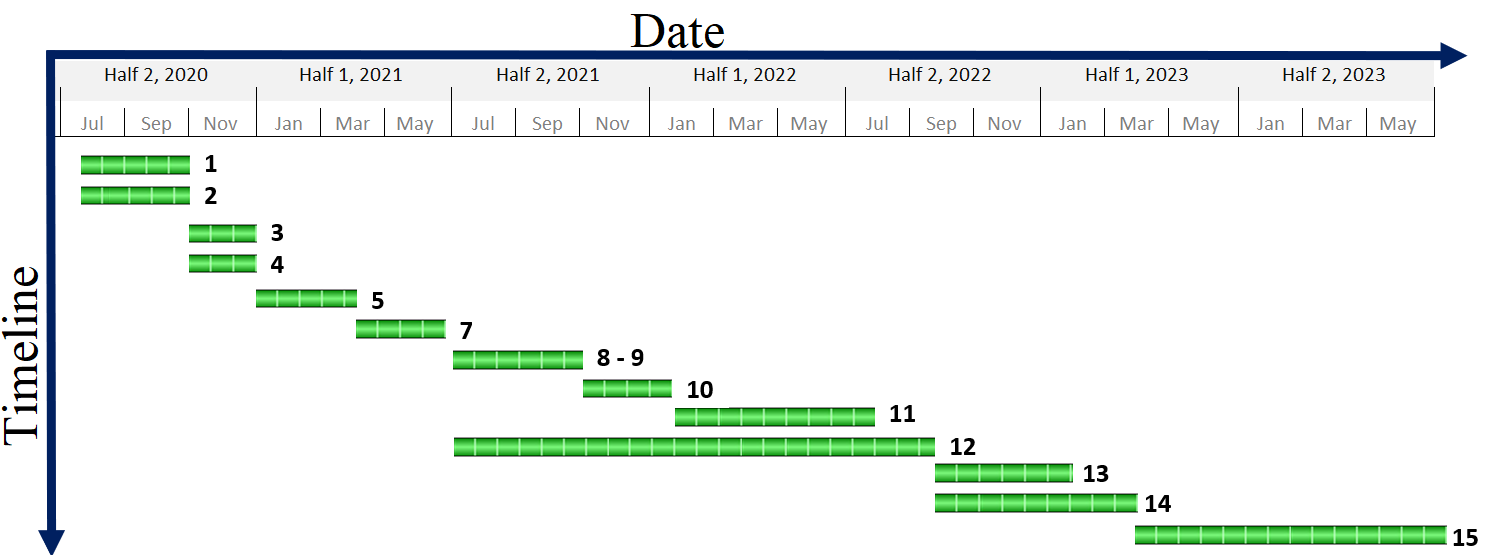


Figure 3: Primavera flow chart for participant timeline

**Data Availability Statement**

The data that support the findings of this study will be available on request from the corresponding author.

**Author Contributions**

All authors had access to research data and contributed to the writing and review of the manuscript.

**Competing Interests**

All authors have disclosed that they have no financial interests or significant relationships with other institutes related to this study or the article.

**Acknowledgements**

The authors are grateful to Oud Al Touba diagnostic and screening centre staff for their contributions to this study.

# 4. References

1. Khan, M.A.B., et al., *Epidemiology of type 2 diabetes–global burden of disease and forecasted trends.* Journal of epidemiology and global health, 2020. **10**(1): p. 107.

2. Zimmet, P., K. Alberti, and J.J.N. Shaw, *Global and societal implications of the diabetes epidemic.* 2001. **414**(6865): p. 782-787.

3. Whiting, D.R., et al., *IDF diabetes atlas: global estimates of the prevalence of diabetes for 2011 and 2030.* 2011. **94**(3): p. 311-321.

4. Ogurtsova, K., et al., *IDF Diabetes Atlas: Global estimates for the prevalence of diabetes for 2015 and 2040.* 2017. **128**: p. 40-50.

5. Rashid Nazir, S.U., et al., *Medication management program: adherence, disease-related knowledge, health-related quality of life, and glycemic control for type 2 diabetes mellitus.* Alternative Therapies in Health & Medicine, 2020. **26**.

6. Hartz, J., L. Yingling, and T.M. Powell-Wiley, *Use of mobile health technology in the prevention and management of diabetes mellitus.* Current cardiology reports, 2016. **18**: p. 1-11.

7. Papakonstantinou, E., et al., *Effects of diet, lifestyle, chrononutrition and alternative dietary interventions on postprandial glycemia and insulin resistance.* Nutrients, 2022. **14**(4): p. 823.

8. Saslow, L.R., et al., *An online intervention comparing a very low-carbohydrate ketogenic diet and lifestyle recommendations versus a plate method diet in overweight individuals with type 2 diabetes: a randomized controlled trial.* Journal of medical Internet research, 2017. **19**(2): p. e36.

9. Boysen, S.R., *Fluid and electrolyte therapy in endocrine disorders: diabetes mellitus and hypoadrenocorticism.* Veterinary Clinics of North America: Small Animal Practice, 2008. **38**(3): p. 699-717.

10. Golden, S.H., et al., *The case for diabetes population health improvement: evidence-based programming for population outcomes in diabetes.* Current diabetes reports, 2017. **17**: p. 1-17.

11. Pérez-Ferre, N., et al., *A Telemedicine system based on Internet and short message service as a new approach in the follow-up of patients with gestational diabetes.* Diabetes research and clinical practice, 2010. **87**(2): p. e15-e17.

12. Levey, A.S., et al., *K/DOQI clinical practice guidelines for chronic kidney disease: evaluation, classification, and stratification.* American Journal of Kidney Diseases, 2002. **39**(2 SUPPL. 1): p. i-ii+.

13. Naljayan, M., et al., *Hypomagnesemia and hypokalemia: a successful oral therapeutic approach after 16 years of potassium and magnesium intravenous replacement therapy.* Clinical kidney journal, 2014. **7**(2): p. 214-216.

14. Satish, R. and G. Gokulnath, *Serum magnesium in recovering acute renal failure.* Indian journal of nephrology, 2008. **18**(3): p. 101.

15. Knezevic, M., *Estimating the long-term costs of diabetic kidney disease: an economic approach.* Applied Economics Letters, 2009. **16**(10): p. 1059-1064.

16. Chong, M.T., *Pharmacist interventions in improving clinical outcomes in patients with type 2 diabetes mellitus among the underrepresented population: a collaborative ambulatory care pharmacy practice (CAPP) approach.* Journal of Research in Pharmacy Practice, 2020. **9**(1): p. 3.

17. Chow, E.P. and A. Hassali, *Medication Counseling Beyond Instituitional: Impact Of Pharmacist-Led Home Medication Review In Type 2 Diabetes Patients.* Value in Health, 2014. **17**(7): p. A746.

18. Steventon, A., et al., *Effect of telehealth on use of secondary care and mortality: findings from the Whole System Demonstrator cluster randomised trial.* Bmj, 2012. **344**.

19. Alasaarela, E. and N.S. Oliver, *Wireless solutions for managing diabetes: a review and future prospects.* Technology and Health Care, 2009. **17**(5-6): p. 353-367.

20. Turton, J., et al., *An evidence‐based approach to developing low‐carbohydrate diets for type 2 diabetes management: A systematic review of interventions and methods.* Diabetes, Obesity and Metabolism, 2019. **21**(11): p. 2513-2525.

21. Sainsbury, E., et al., *Effect of dietary carbohydrate restriction on glycemic control in adults with diabetes: a systematic review and meta-analysis.* Diabetes Research and Clinical Practice, 2018. **139**: p. 239-252.

22. Snorgaard, O., et al., *Systematic review and meta-analysis of dietary carbohydrate restriction in patients with type 2 diabetes.* BMJ Open Diabetes Research and Care, 2017. **5**(1): p. e000354.

23. van Zuuren, E.J., et al., *Effects of low-carbohydrate-compared with low-fat-diet interventions on metabolic control in people with type 2 diabetes: a systematic review including GRADE assessments.* The American journal of clinical nutrition, 2018. **108**(2): p. 300-331.

24. Watson, N., et al., *Effects of low-fat diets differing in protein and carbohydrate content on cardiometabolic risk factors during weight loss and weight maintenance in obese adults with type 2 diabetes.* Nutrients, 2016. **8**(5): p. 289.

25. Tay, J., et al., *A very low-carbohydrate, low–saturated fat diet for type 2 diabetes management: a randomized trial.* Diabetes care, 2014. **37**(11): p. 2909-2918.

26. Grover, A. and A. Joshi, *An overview of chronic disease models: a systematic literature review.* Global journal of health science, 2015. **7**(2): p. 210.

27. Clement, M., et al., *Organization of diabetes care.* Canadian journal of diabetes, 2013. **37**: p. S20-S25.

28. Kopple, J.D., et al., *Nutritional status of patients with different levels of chronic renal insufficiency.* Kidney International Supplement, 1989(27).

29. Wiggins, K.L. and K.S. Harvey, *A review of guidelines for nutrition care of renal patients.* Journal of Renal Nutrition, 2002. **12**(3): p. 190-196.

30. Hoss, S., et al., *Serum potassium levels and outcome in patients with chronic heart failure.* The American journal of cardiology, 2016. **118**(12): p. 1868-1874.

31. Weinberger, M.H., *Sodium, potassium, and blood pressure.* American journal of hypertension, 1997. **10**(S4): p. 46S-48S.

32. Mahboob, S., *Electrolytes and Na-K-ATPase: potential risk factors for the development of diabetic nephropathy.* Pak. J. Pharm. Sci, 2008. **21**(2): p. 172-179.

33. Reza, M.E., et al., *Serum and intracellular levels of ionized sodium, potassium, and magnesium in type 2 diabetic subjects.* International Journal of Nutrition, Pharmacology, Neurological Diseases, 2015. **5**(2): p. 69.

34. Larsson, S.C. and A. Wolk, *Magnesium intake and risk of type 2 diabetes: a meta‐analysis.* Journal of internal medicine, 2007. **262**(2): p. 208-214.

35. Ito, H., et al., *Disorders of fluid and electrolyte metabolism in elderly diabetics.* Nihon Ronen Igakkai zasshi. Japanese Journal of Geriatrics, 1989. **26**(3): p. 233-239.

36. Shashidhar, K.N., et al., *Hypermagnesemia in diabetic end stage renal disease (ESRD) patients.* Indian Journal of Clinical Biochemistry, 2007. **22**(2): p. 164.

37. Nilsson, E., et al., *Incidence and determinants of hyperkalemia and hypokalemia in a large healthcare system.* International journal of cardiology, 2017. **245**: p. 277-284.

38. Kovesdy, C.P., et al., *Serum potassium and adverse outcomes across the range of kidney function: a CKD Prognosis Consortium meta-analysis.* European heart journal, 2018. **39**(17): p. 1535-1542.

39. Disease, K. and I.G.O.J.K.I. Suppl, *Chapter 1: Definition and classification of CKD.* 2013. **3**(1): p. 19-62.

40. Association, A.D.A.J.C.d.a.p.o.t.A.D., *Standards of medical care in diabetes—2016 abridged for primary care providers.* 2016. **34**(1): p. 3.

41. Awwad, O., et al., *Translation and validation of the Arabic version of the Morisky, Green and Levine (MGL) adherence scale.* PloS one, 2022. **17**(10): p. e0275778.

42. Kozma, C.M., et al., *Medication possession ratio: implications of using fixed and variable observation periods in assessing adherence with disease-modifying drugs in patients with multiple sclerosis.* Patient preference and adherence, 2013: p. 509-516.

43. Hedegaard, U., et al., *Improving medication adherence in patients with hypertension: a randomized trial.* The American journal of medicine, 2015. **128**(12): p. 1351-1361.

44. Sperber, C.M., S.R. Samarasinghe, and G.P. Lomax, *An upper and lower bound of the medication possession ratio.* Patient preference and adherence, 2017: p. 1469-1478.

45. Tang, K.L., H. Quan, and D.M. Rabi, *Measuring medication adherence in patients with incident hypertension: a retrospective cohort study.* BMC health services research, 2017. **17**: p. 1-16.

46. Rolnick, S.J., et al., *Patient characteristics associated with medication adherence.* Clinical medicine & research, 2013. **11**(2): p. 54-65.

47. Choudhry, N.K., et al., *Measuring concurrent adherence to multiple related medications.* The American journal of managed care, 2009. **15**(7): p. 457.

48. Steiner, J.F., et al., *A general method of compliance assessment using centralized pharmacy records: description and validation.* Medical care, 1988: p. 814-823.

49. Morisky, D.E., L.W. Green, and D.M. Levine, *Concurrent and predictive validity of a self-reported measure of medication adherence.* Medical care, 1986: p. 67-74.

50. (SEHA), A.D.H.S.C. *Seha App*. 2024 Oct 19, 2023 [cited 2024 14-01-2024]; 12.3.1:[SEHA Mobile Application]. Available from: <https://www.seha.ae/sehaApp>.

51. Toobert, D.J., S.E. Hampson, and R.E. Glasgow, *The summary of diabetes self-care activities measure: results from 7 studies and a revised scale.* Diabetes care, 2000. **23**(7): p. 943-950.

52. Toobert, D.J., S.E. Hampson, and R.E.J.D.c. Glasgow, *The summary of diabetes self-care activities measure: results from 7 studies and a revised scale.* 2000. **23**(7): p. 943-950.

53. Prevention, U.A.E.M.o.H.a. *Calories Calculation*. 2022; Available from: <https://mohap.gov.ae/en/more/awareness-center/calories-calculation>.

54. Requeriments, H.E., *Report of a joint FAO/WHO/UNU expert consultation*. 2001, Rome.

55. Camelon, K.M., et al., *The Plate Model: a visual method of teaching meal planning.* Journal of the American Dietetic Association, 1998. **98**(10): p. 1155-1158.

56. Haskell, W.L., et al., *Physical activity and public health: updated recommendation for adults from the American College of Sports Medicine and the American Heart Association.* Circulation, 2007. **116**(9): p. 1081.

57. Cowie, A., et al., *Standards and core components for cardiovascular disease prevention and rehabilitation.* Heart, 2019. **105**(7): p. 510-515.

58. Borg, G., *The Borg CR10 Folder. A Method for Measuring Intensity of Experience*. 2004, Stockholm, Sweden: Borg Perception.

59. *British Association for Clinical Pharmacy Research (BACPR). (2019, April). BACPR annual report. Retrieved from [*[*https://www.bacpr.org/__data/assets/pdf_file/0008/60110/BACPR-Ref-Table-Booklet-April-2019.pdf*](https://www.bacpr.org/__data/assets/pdf_file/0008/60110/BACPR-Ref-Table-Booklet-April-2019.pdf)*]*.

60. Ainsworth, B.E., et al., *Compendium of physical activities: an update of activity codes and MET intensities.* Medicine and science in sports and exercise, 2000. **32**(9; SUPP/1): p. S498-S504.

61. Verbalis, J.G., et al., *Diagnosis, evaluation, and treatment of hyponatremia: expert panel recommendations.* The American journal of medicine, 2013. **126**(10): p. S1-S42.

62. Sterns, R.H., *Disorders of plasma sodium.* The New England Journal of Medicine, 2015. **372**(13): p. 1269-1269.

63. Intakes, I.o.M.S.C.o.t.S.E.o.D.R., *Dietary reference intakes for calcium, phosphorus, magnesium, vitamin D, and fluoride.* 1997.

64. Mount, D.B.J.U.W., *Clinical manifestations and treatment of hypokalemia in adults.* 2017.

65. Viera, A.J. and N.J.A.f.p. Wouk, *Potassium disorders: hypokalemia and hyperkalemia.* 2015. **92**(6): p. 487-495.

66. Yu, A. and A.J.U.W. Gupta, MA: UpToDate, *Hypermagnesemia: Causes, symptoms, and treatment.* 2020.

67. Cohn, J.N., et al., *New guidelines for potassium replacement in clinical practice: a contemporary review by the National Council on Potassium in Clinical Practice.* 2000. **160**(16): p. 2429-2436.

68. Kraft, M.D., et al., *Treatment of electrolyte disorders in adult patients in the intensive care unit.* 2005. **62**(16): p. 1663-1682.

69. Georgiev, G.Z., *Sample size calculator.* URL: <https://www.gigacalculator.com/calculators/power-sample-size-calculator.php>, 2020.
